# Effect of Stockholm Convention Listing on Temporal Trends of Halogenated Flame Retardants in Herring Gull Eggs in Canada (2008–2023)

**DOI:** 10.1007/s00244-025-01173-2

**Published:** 2026-02-02

**Authors:** H. L. Vanderlip, K. D. Hughes, D. M. Orihel, V. L. Friesen, S R de Solla, R. J. Letcher, P. A. Martin, R. A. Lavoie, M. L. Eng, J. F. Provencher

**Affiliations:** 1 Department of Biology, Queen’s University, Kingston, ON Canada; 2 Broadwing Biological Consulting, Port Perry, ON Canada; 3 School of Environmental Studies, Queen’s University, Kingston, ON Canada; 4 Ecotoxicology and Wildlife Health Division, Science and Technology Branch, Environment and Climate Change Canada, Burlington, ON Canada; 5 Ecotoxicology and Wildlife Health Division, Science and Technology Branch, Environment and Climate Change Canada, Ottawa, ON Canada; 6 Ecotoxicology and Wildlife Health Division, Science and Technology Branch, Environment and Climate Change Canada, Québec, QC Canada; 7 Ecotoxicology and Wildlife Health Division, Science and Technology Branch, Environment and Climate Change Canada, Dartmouth, NS Canada

## Abstract

Halogenated flame retardants (HFRs) are chemical additives used in many household and commercial products. Due to their persistence, long-range transport, bioaccumulative potential, and toxicity, several HFRs were listed for elimination or restrictions under the international Stockholm Convention on Persistent Organic Pollutants (SC-POPs). To understand how levels of HFRs in the environment have changed since being listed under the SC-POPs, herring gull (*Larus argentatus*) eggs were collected from 17 colonies across Canada (Laurentian Great Lakes, Niagara and St. Lawrence rivers, and Atlantic and Arctic regions) from 2008–2023. Eggs were analyzed for SC-POPs-listed HFRs; total-α-hexabromocyclododecane (HBCDD), Σpolybrominated diphenyl ethers (PBDEs; sum concentration of 11 tri- to hepta-BDE congeners), BDE-209, and ΣDechlorane Plus (DP; sum concentration of *syn-* and *anti-*isomers). Overall, no significant temporal changes in concentrations of the four target classes of HFRs were found for most comparisons across colonies (75%, 51/68). Significant declines were found for 13% of class-colony comparisons while BDE-209 concentrations increased significantly at one colony. Evidence of a significant influence of the SC-POPs on concentrations was equivocal, with 6% of trends declining with the year of nomination, year of listing, or before the year of nomination, and 4% of trends showing increases. Despite more than a decade of regulation for some of these compounds, herring gulls continue to be exposed to HFRs, presumably from sources such as stockpiles, discarded products, or re-release from environmental reservoirs. Continued multi-site monitoring of HFRs is required to fully assess the effectiveness of regulations that may take several decades to show consistent evidence of reduced exposure in gulls.

**Supplementary Information:**

The online version contains supplementary material available at 10.1007/s00244-025-01173-2.

## Introduction

Halogenated flame retardants (HFRs) are a class of chemicals used as additives in a wide range of polymeric products to reduce flammability and comply with fire safety standards (de Wit 2002; United Nations Environmental Program [UNEP], 2023a). Because many HFRs are additive rather than chemically bound to a product, these chemicals can be released into the environment during their production, incorporation into products, use, and disposal (Kefeni et al. 2011). Urban environments, landfills, and wastewater treatment facilities are large sources of HFRs into the environment (Kefeni et al. 2011; Akortia et al. 2016; Tongue et al. 2019). Many HFRs are considered to be persistent organic pollutants (POPs) given their bioaccumulative nature, potential for long-range environmental transport, environmental persistence, and adverse effects on human health and/or the environment (UNEP, 2023a).

The Stockholm Convention on POPs (hereafter “Stockholm Convention”) is a UNEP treaty that was created in response to global concern about POPs and seeks to protect human and environmental health from POPs (UNEP, 2023a). This treaty provides a framework for control, reduction and/or elimination in production and use of POPs and for their regulation in countries that have ratified the treaty. Among the POPs listed under Annex A (in which parties must take measures to eliminate the production and use of listed chemicals) of the Stockholm Convention are polybrominated diphenyl ethers (PBDEs) that include tetra-BDEs and penta-BDEs present in the commercial penta-BDE mixture, and hexa-BDEs and hepta-BDEs, which are the main components of the commercial octa-BDE mixture. While commercial penta- and octa-BDEs were nominated separately in 2005 and 2006, respectively, the main components of these mixtures were listed under the Stockholm Convention in 2009 (UNEP, 2009). Other POPs include hexabromocyclododecane (HBCDD) that was nominated in 2008 and listed for elimination in 2013 (UNEP, 2013); deca-BDE (BDE-209, present in the commercial c-deca-BDE mixture) that was nominated in 2013 and listed in 2017 (UNEP, 2017); and, most recently, Dechlorane Plus (DP; includes *syn*- and *anti*-isomers) that was nominated in 2019 and listed in 2023 with specific exemptions (UNEP 2023b). Given that the purpose of SC-POPs is to reduce emissions into the environment through national regulations made by the signatory parties, success of the treaty can be assessed by determining if chemical concentrations in environmental matrices have declined following the nomination and listing of these chemicals to Annex A.

Herring gull (*Larus argentatus* = *L. smithsonianus*; hereafter, ‘herring gull’) eggs have been used for decades to monitor concentrations of POPs, including quantifiable HFRs, in Canadian environments (Su et al. 2015). Eggs can be taken without reducing population productivity as females often replace the sampled egg. The overall high trophic position and long lifespan of herring gulls make them particularly useful as a sentinel bioindicator of biomagnification and bioaccumulation of contaminants (Furness and Camphuysen 1997; Hebert et al. 1999). Additionally, the extensive geographic distribution of herring gulls allows for regional comparisons of contaminant trends over their foraging range at nesting colonies across Canada (Furness and Camphuysen 1997; Anderson et al. 2019). During the breeding season, foraging by breeding adults are limited to areas a few kms from their nests; 50% of the foraging distances of herring gulls in the New England states were 8 km or less, and 75% were 16 km or less (Birds of the World; citing Drury and Nisbet, 1972). Similarly, for marine and mainland feeding herring gulls, Enners et al. (2018) reported mean maximum distances (e.g. the mean longest distance between a foraging location and the gull nest) of 4.2, 6.8 and 9.2 km for breeding herring gulls from three different colonies in the Wadden Sea in Germany. We do not have specific foraging ranges around colonies we monitored, but these published estimates are likely broadly representative of typical foraging ranges of herring gulls.

Our study was designed to test the hypothesis that concentrations of a halogenated flame retardant in the environment decline in response to the listing of the chemical under the SC-POPs. Using the eggs of herring gulls as biomonitors, temporal trends of HFRs, including PBDEs, HBCDD, and DP, were investigated in Canadian ecosystems between 2008 to 2023. Based on when PBDEs and HBCDD were listed under the Stockholm Convention (~ 2009 and 2013, respectively), our prediction is that the monitoring data will show that concentrations of these chemicals in herring gull eggs declined between 2008 and 2023 with changes in the trajectories corresponding near the years when the compounds were listed or nominated to the SC-POPs. However, we do not expect to see declines in DP over this same period, given this chemical was not listed until 2023.

Lastly, we determined if HFR concentrations varied among regions (Great Lakes + Niagara River, St. Lawrence River, Atlantic and Arctic). We hypothesized that HFR concentrations would be elevated in eggs from the Great Lakes-St. Lawrence River basin, given the greater population and industry in central-eastern Canada. Conversely, HFR concentrations would be lower in eggs from the Arctic and Atlantic regions.

## Methods

### Sampling Methods

**Fig. 1:**
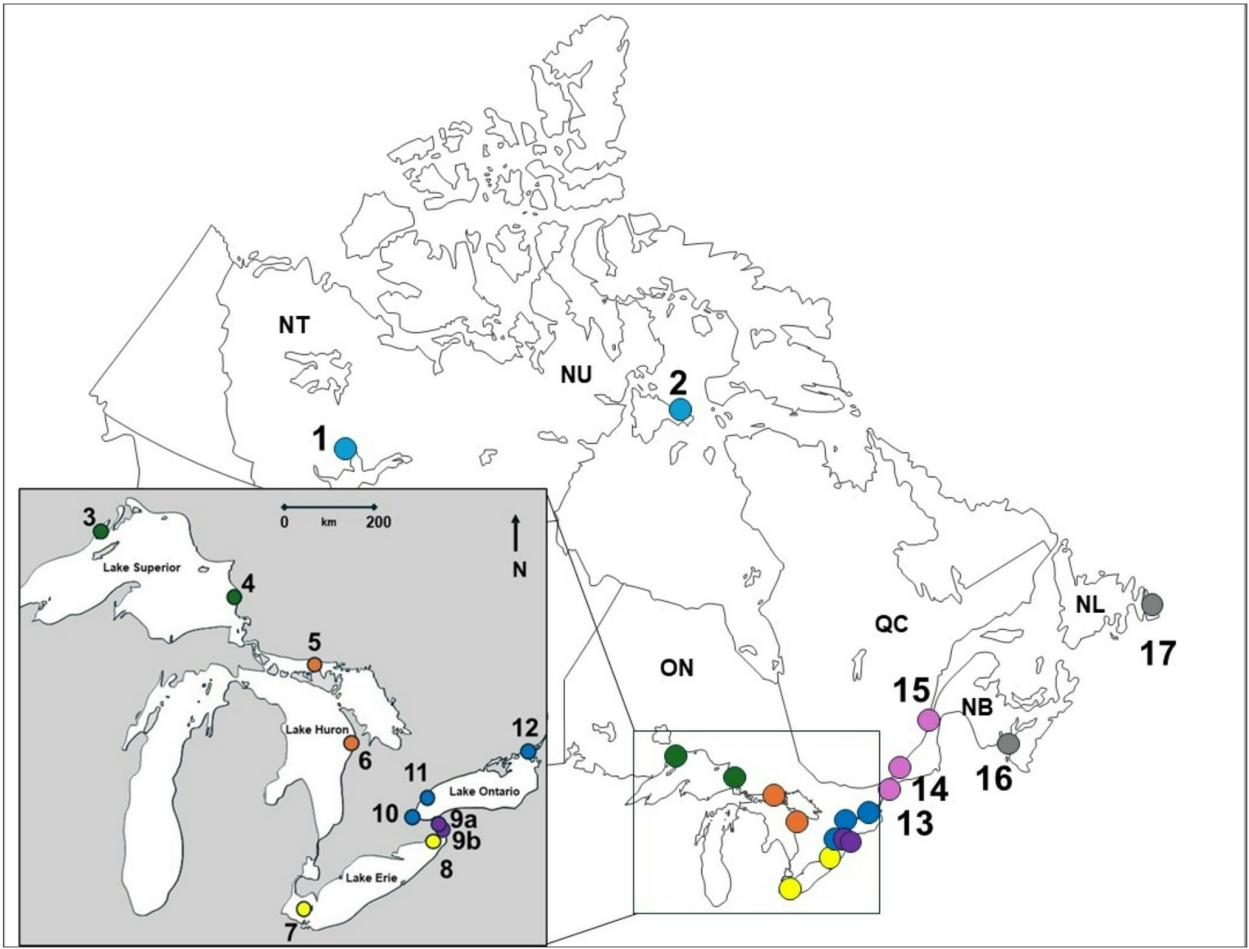
Locations of herring gull colonies where eggs were collected in Canada from 2008–2023 with the Laurentian Great Lakes shown in the inset. 1: Great Slave Lake, 2: East Bay, 3: Granite Island, 4: Agawa Rocks, 5: Double Island, 6: Chantry Island, 7: Middle Island, 8: Port Colborne, 9a and 9b: Weseloh Rocks and Buffalo Harbor, respectively (considered one colony), 10: Hamilton Harbour, 11: Toronto Harbour, 12: Snake Island, 13: Strachan Island, 14: Île Deslauriers, 15: Île Bellechasse, 16: Kent Island, and 17: Gull Island. Colours refer to the region or waterbody in which colonies are found. Colony regions and colours from left (west) to right (east): Arctic region (light blue), Lake Superior (green), Lake Huron (orange), Lake Erie (yellow), Niagara River (purple), Lake Ontario (dark blue), St. Lawrence River (pink), and Atlantic coastal region (grey)

Herring gull eggs were collected from 17 colonies across Canada (Fig. 1), spanning the Laurentian Great Lakes (n = 9 colonies), Niagara River (n = 1), St. Lawrence River (n = 3), and Atlantic and Arctic regions (n = 2 for each). The colonies span a latitude from 41.682 degrees (Middle Is., Ontario) to 64.029 degrees (East Bay, Nunavut) and a longitude from -115.526 degrees (Great Slave Lake, Northwest Territories) to -52.772 degrees (Gull Is., Newfoundland and Labrador).

Eggs were collected annually from colonies from 2008 to 2023 under Environment and Climate Change Canada’s (ECCC’s) Chemicals Management Plan’s Wildlife Monitoring and Surveillance program and the Great Lakes Herring Gull Contaminant Monitoring Program (Supplementary Information, Table S1); one exception was for the two Arctic colonies where egg collections were initiated in 2010. Eggs from colonies sampled under ECCC’s Chemicals Management Plan were chemically analyzed for HFRs in every year from 2008–2012, and then in alternate years thereafter. Eggs from Great Lakes colonies were analyzed for HFRs every year, where possible. For the colony site on the Niagara River, eggs were collected from Weseloh Rocks in Ontario from 2008–2015 and then from Buffalo Harbor situated in upper New York State (United States) in 2018 and 2019 since the colony at Weseloh Rocks was inaccessible due to high water levels; these two colonies are near each other and were treated as one site, hereafter known as Weseloh Rocks. The total numbers of years that each colony was analyzed for HFRs ranged from 6 to 16 years.

Each year, colonies in the Great Lakes, Niagara and St. Lawrence rivers, and Atlantic regions were sampled between late April to early May, and Arctic colonies were sampled in June, based on the timing of completion for egg laying. At each colony, a single egg was haphazardly selected from each of 10–15 nests with completed clutches of three eggs. Mass (g), length (cm), and breadth (cm) of sampled eggs were measured during annual processing of eggs, and volume was calculated according to Ryder (1975; *egg volume* = *kld*^*2*^, *k* = 0.489, *l* = length, *d* = breadth). Egg measurements were recorded for collections from 2008 to 2019 only. Eggs were refrigerated until they were sent to the National Wildlife Research Centre (NWRC) in Ottawa, Ontario, Canada.

At the NWRC, whole egg contents (yolk and albumen) were homogenized using an electric mixer and pooled on an equal wet weight basis. Pool sizes ranged from 2 to 15 eggs and the number of pools analyzed varied between one and five per colony in each year. Homogenates were stored at -40 °C until samples were sent to the NWRC for chemical analysis. In 2008, ten eggs from Gull Is., Île Bellechasse, Île Deslauriers, and Kent Is. were homogenized and then chemically analyzed as individual eggs. The results of these analyses were randomly paired and averaged to approximate five egg pools (replicates) to match with other sites in other years.

### Chemical Analysis of Target HFRs in Eggs

To measure all target HFRs in eggs, samples were homogenized with either anhydrous sodium sulfate or diatomaceous earth and spiked with internal standards followed by neutral extraction with DCM:Hexane (1:1). Lipids and biogenic materials were removed from the extract by gel permeation chromatography with further lipid removal and cleanup using solid phase extraction.

Details of chemical analyses are in Smythe et al. (2020), with references included therein; a truncated version of the methods follows. Purified sample extracts were analyzed for HFRs using a capillary gas chromatograph coupled with a mass selective detector operated in selected ion monitoring mode. Samples were analyzed for α-HBCDD, 13 PBDE congeners (BDE-17, -28, -47, -49, -66, -85, -99, -100, -138, -153, -183, -190 and -209), and *syn*- and *anti*-DP. HBCDD was quantified as total-α-HBCDD as β- and γ-HBCDD residues are thermally isomerized to α-HBCDD when temperatures exceed 160 °C. Analytical results were recovery corrected using at least two of the following internal standards run in each year of chemical analysis: BDE-30, BDE-118, BDE-156, ^13^C_10_-*syn*-DP, ^13^C_10_-*anti*-DP, and ^13^C_12_-BDE-209. Chemical standards were obtained from Wellington Laboratories (Guelph, Ontario, Canada). Standard reference materials were simultaneously run with each batch of samples to ensure accuracy; these included National Institute of Standards and Technology (NIST) Standard Reference Material®1947 (Lake Michigan fish tissue) and double-crested cormorant (*Nannopterum auritum*) in-house reference material (egg pool), the latter of which was used exclusively after 2021 when NIST Standard Reference Material®1947 was no longer available for purchase. Where possible, a duplicate extraction and injection was selected in order to monitor extraction and instrumental precision. Samples were also corrected for background contamination using method blanks that were processed through the entire extraction and analysis method. Data were verified to ensure that all quality criteria were met and data outside of specified QA/QC criteria were flagged (e.g. the relative percent difference (RPD) of replicate in-house reference material were within control limits), with repeat chemical analysis in cases where criteria were notably violated. The mean RPD was 6.43, with a RPD limit of 20; 99.1% of replicates were within control limits. The mean % recovery of HFRs was 99.6% (range = 78.5–120%).

In 2008 when the study was initiated, the two DP isomers were not included in the list of analytes for chemical analysis. As such, a second set of chemical analyses was conducted to include *syn*- and *anti*-DP as analytes (and other HFRs, not reported here) for egg samples from colonies monitored under the Chemicals Management Plan (n = 20) and on the Great Lakes (n = 11). Chemical analyses of BDE congeners and/or HBCDD in eggs were also conducted at this time for comparisons of concentrations in the first set of analyses of 2008 eggs and mean concentrations were determined between the two sets where possible. All concentrations are reported as ng/g wet weight.

Method limits of detection (MLODs) for target HFRs were generally between 0.01 and 1.0 ng/g and varied by congener and year (Supplementary Information, Table S2); MLODs for BDE-209 were notably higher in three years of analysis and equal to 5.0 ng/g. Where available, MLODs were based on a signal to noise ratio of 3, and method limits of quantification (MLOQ) were based on a signal to noise ratio of 10, relative to the standard deviation of the blanks. Although MLODs and MLOQs of HFRs differed among years, the proportions of samples with HFR concentrations above MLODs or MLOQs, i.e., with reportable concentrations, were generally similar among years. Of the 511 egg samples analyzed for HFRs, at least 84% exceeded MLODs or MLOQs for individual BDE congeners, HBCDD and *anti*-DP, with a few exceptions (Supplementary Information, Table S2). *Syn-*DP had detection rates that were lower and more variable among years (61 – 95%).

### Statistical Data Analysis

To analyze trends of the commercial penta- and octa-BDE mixtures, lower brominated BDE congeners (BDE-17 through BDE-183) were summed (hereafter referred to as Σ_11_PBDE). BDE-190 was not included in ΣPBDEs since it was detected in less than 3% of all analyzed samples. BDE-209 was analyzed separately as a proxy for commercial deca-BDEs since these mixtures are comprised of > 90% BDE-209 (La Guardia et al. 2006). Dechlorane Plus was analyzed as the sum of the *syn-* and *anti*-isomers, hereafter referred to as ΣDP.

To account for concentrations that were below MLODs or MLOQs in our statistical analysis, for each HFR (which includes each individual BDE congener), the percentage of egg samples with reportable concentrations was first determined at each colony. When more than 50% of samples had reportable HFR concentrations, maximum likelihood estimation was used to generate replacement values (Helsel 2012). Using Excel’s (Microsoft Corp) iterative Solver function, HFR observations that were below detection limits were replaced with values that fit along a log-transformed quantile-normal plot of the population mean and variance (de Solla et al. 2012). Replacement values are unique and were ranked according to concentrations of other reference HFRs that were frequently detected. BDE-99 and *syn*-DP were used as the reference HFRs for estimates of individual PBDE congeners and other HFRs (i.e., HBCDD and *anti-*DP), respectively, since highly significant correlations were generally found between concentrations of these compounds in eggs. When fewer than 50% of samples had reportable HFR concentrations at a colony, no replacement value was generated and a zero concentration was given for the sample.

For the four target classes of HFRs (Σ_11_PBDE, BDE-209, HBCDD, ΣDP), arithmetic means were calculated for each collection year in cases where multiple egg pools were analyzed, and single egg pool values were used when a single sample was analyzed. Previous work has demonstrated that the contaminant concentration in a single herring gull egg pool is equivalent to the mean concentration when eggs are analyzed individually (Turle and Collins 1992). Due to the general skewness in distributions of HFR concentrations, mean concentrations were natural log transformed to meet conditions of normality for residuals at each colony. Linear correlation analysis (Pearson r) was conducted between mean concentrations of the four target HFRs and mean egg mass, volume, and percent lipid at each colony in each study year; if conditions for normality of residuals were not met, a Spearman rank correlation (r_s_) was conducted.

Since only seven significant relationships were found between mean egg volume or mean egg mass and mean HFR concentrations at 17 colonies and given that most comparisons (95% or 129/136) were not related to egg size, we did not use egg size as covariates or factors in models. Significant relationships were largely not evident for correlations between mean percent lipid and mean HFR concentrations in eggs for most class-colony comparisons (99% or 67/68). The single exception was for ∑DP, where a significant positive relationship was found with egg percent lipid at Double Island (r_s_ = 0.73, *p* = 0.003, N = 14) only. Further, when we compared the full models (including lipids as a predictor) with the reduced model (excluding lipids) using log-likelihood tests, inclusion of lipids did not significantly improve predictions of HFRs (Supplemental Information, *Contribution of lipids to models predicting HFR concentrations*, and Table S3). Hence, we did not include lipid as a variable in our models.

Details of mean egg volume, mean egg mass and mean percent lipid for each study colony across all study years are provided in Supplementary Information, Table S4.

To determine if temporal patterns of HFR concentrations changed in eggs at study colonies, breakpoint analysis, also known as change point or segmented linear regression was conducted. A breakpoint represents a change in the slope of the model such that it significantly improves the fit of the model to the data. Here, the breakpoint represents the year when there is a significant change in the trajectory of the observed trend in HFR concentrations at a colony. Generalized Linear Models were performed using the R package *segmented* (1.6–4; Muggeo 2003), using a normal error term with identity link function to generate breakpoint regressions.

Following this analysis, we identified five possible trend models to describe the temporal patterns in HFR concentrations in gull eggs: 1) increasing trend (IT): a breakpoint occurred and HFR concentrations increased after the breakpoint; 2) listing improvement (LI): a breakpoint occurred after the year of listing and HFR concentrations decreased after the breakpoint; 3) nomination improvement (NI): a breakpoint occurred after the year of nomination and HFR concentrations decreased after the breakpoint; 4) early improvement (EI): a breakpoint occurred before the year of nomination and HFR concentrations decreased after the breakpoint; and 5) consistent trend (CT): no breakpoint occurred and the result of linear regression analysis, if significant, is presented.

We also determined if HFR concentrations varied among colonies based on Region. Each colony was grouped by Region (Great Lakes + Niagara River, St. Lawrence River, Atlantic and Arctic). We used a Linear Mixed-Effects Model to determine the effect of Region on HFRs, using natural log transformed contaminant concentrations. We used likelihood‐ratio tests comparing the full models with the null models (with or without Region as a factor) to determine if including Region significantly improved the prediction of HFR concentrations in eggs. For all HFRs, the models were fit using the R package *lmerTest*/lme4, with Region as a fixed effect and Colony (nested within Region) and Year as random effects; hence:

$${y}_{ijk}={\beta }_{0}+{\beta }_{Region(i)}+{u}_{ij}+{v}_{k}+{\epsilon }_{ijk}$$

where *y*_*ijk*_ = ln[HFR] for colony *j* in Region *i* during Year *k*; β_0_ = overall intercept, β_*Region*(*i*)_ = fixed effect for Region *i*, *u*_*ij*_ = random intercept for colony *j* (nested within Region *i*), *v*_*k*_ = random intercept for Year *k*, and *ε*_*ijk*_ = residual error. We used sum-to-zero contrasts, and thus the intercept is the overall mean concentration among Regions. Satterthwaite’s approximation for degrees of freedom was used for tests. The R package *emmeans* was used to calculate estimated marginal means that were back transformed to the original scale, to compare mean concentrations for each Region to the overall contaminant mean.

Data analyses were conducted in Excel version 2409 (Microsoft Corp), Statistica for Windows Version 7 (StatSoft, Inc.), and R versions 4.2.1 and 4.2.3 (R Core Team, 2023), and we used an α = 0.05 as the cutoff for determining significance.

## Results

### HBCDD

**Fig. 2:**
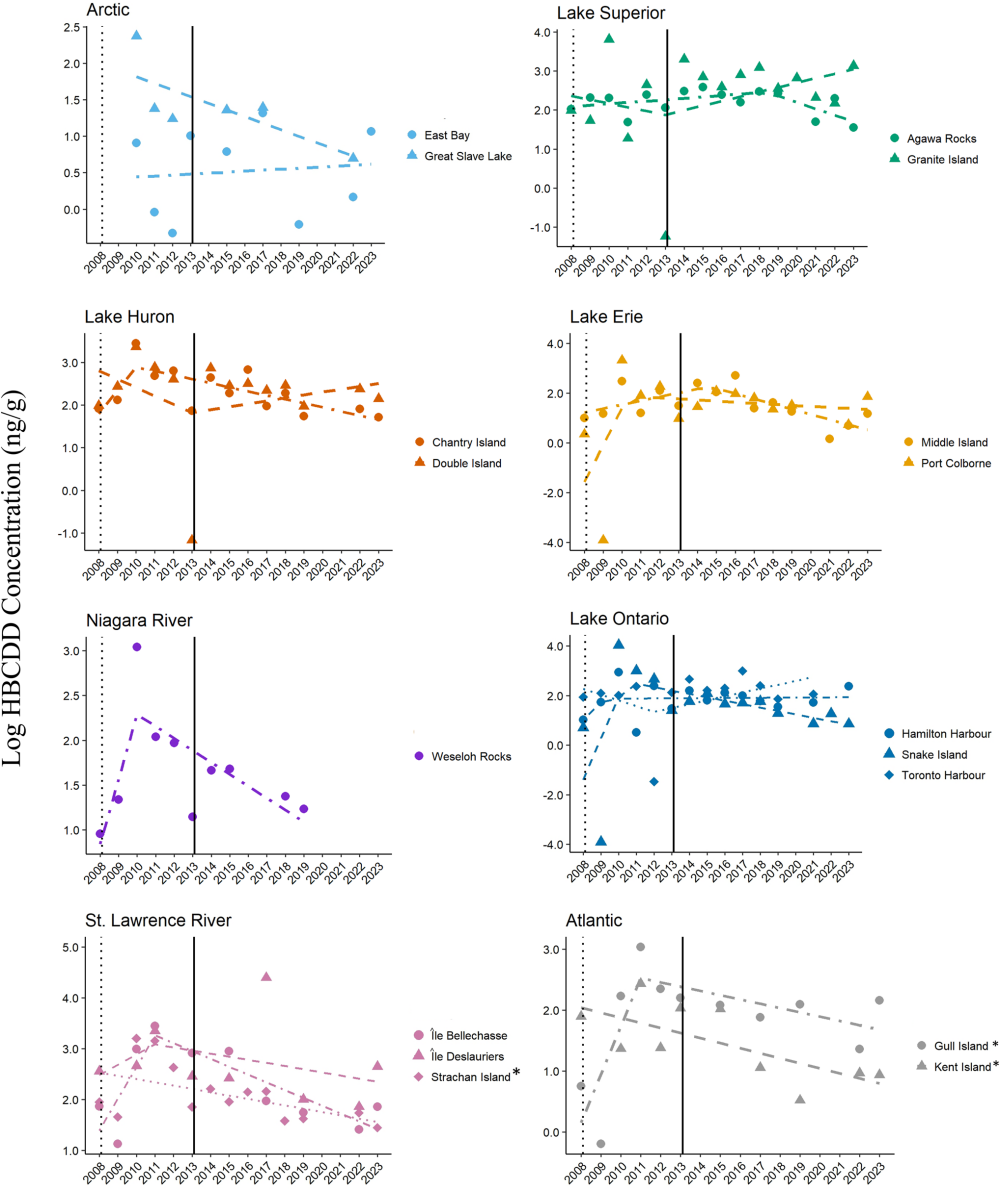
Trends and breakpoints for concentrations (ln ng/g, wet weight) of HBCDD in herring gull eggs from 17 colonies in Canada from 2008–2023. The year of nomination of HBCDD to Annex A of the Stockholm Convention (2008) is shown as a dotted vertical line and the year of listing (2013) is shown as a solid vertical line. Models that are statistically significant are denoted by an asterisk (*) beside the colony name

Significant temporal changes in HBCDD concentrations were evident in eggs from three of 17 herring gull colonies from 2008 to 2023 (Fig. 2; Table 1). Eggs from Gull Island in the Atlantic region followed the nomination improvement (NI) trend model showing an increase of 120% in HBCDD per year (exp(0.79[β]) ng/g/year), a significant breakpoint in 2011, followed by a 7% annual decrease (exp(-0.07[β]) ng/g/year; t = 2.86, *p* < 0.03). This breakpoint year occurred three years after nomination (2008) and two years prior to listing of HBCDD (2013). Consistent trends (CT) in HBCDD concentrations were found in eggs from Kent Island in the Atlantic region and Strachan Island on the St. Lawrence River, with significant declines reported from 2008–2023 (8% decrease or exp(-0.08[β]) ng/g/year and 6% decrease or exp(-0.06[β]) ng/g/year, respectively; t < -2.26, *p* < 0.05). Note that data are shown as log-transformed values with trends plotted on the natural logarithmic scale. Mean concentrations of HBCDD and other HFRs by year and colony are provided in Supplementary Information, Table S5.

**Table 1:** Breakpoint year and slopes (ln ng g^−1^ year^−1^) of generalized linear models for mean (± SE) concentrations of HBCDD, ∑_11_PBDE congeners, BDE-209 and ∑DP in herring gull eggs from 17 colonies in Canada from 2008–2023

Flame Retardant	Region	Colony	No. Years	Breakpoint Year (± SE)	Slope Before Breakpoint (± SE)	Slope After Breakpoint (± SE)	T value	Pr( >|t|)	Trend Model
HBCDD	Arctic	East Bay	9	NA^a^	0.01 (± 0.05)	NA	0.27	0.79	
		Great Slave Lake	6	NA^a^	-0.09 (± 0.04)	NA	2.30	0.08	
	Atlantic	Gull Is	11	2011 (± 0.79)	0.79 (± 0.28)	-0.07 (± 0.06)	2.86	0.025*	NI
		Kent Is	10	NA	-0.08 (± 0.03)	NA	-2.74	0.026*	CT
	Lake Erie	Middle Is	15	2015 (± 1.53)	0.16 (± 0.10)	-0.21 (± 0.07)	1.54	0.15	
		Port Colborne	14	2010 (± 1.26)	1.49 (± 1.09)	-0.04 (± 0.13)	1.36	0.20	
	Lake Huron	Chantry Is	14	2010 (± 1.36)	0.55 (± 0.54)	-0.09 (± 0.028)	1.02	0.33	
		Double Is	14	2013 (± 5.16)	-0.20 (± 0.36)	0.07 (± 0.12)	-0.54	0.60	
	Lake Ontario	Hamilton H	14	2009 (± 1.22)	0.71 (± 0.89)	0.004 (0.05)	0.79	0.45	
		Snake Is	15	2010 (± 1.11)	1.67 (± 1.12)	-0.14 (± 0.12)	1.49	0.17	
		Toronto H	13	2012 (± 4.01)	-0.23 (± 0.52)	0.16 (± 0.14)	-0.46	0.66	
	Lake Superior	Agawa Rocks	15	2018 (± 1.71)	0.04 (± 0.03)	-0.17 (± 0.09)	1.34	0.21	
		Granite Is	16	2013 (± 6.80)	-0.10 (± 0.39)	0.12 (± 0.12)	-0.25	0.81	
	Niagara R	Weseloh Rocks	10	2010 (± 1.24)	0.72 (± 0.65)	-0.13 (± 0.05)	-1.12	0.31	
	St. Lawrence R	Île Bellechasse	10	2011 (± 1.15)	0.62 (± 0.38)	-0.15 (± 0.05)	1.65	0.15	
		Île Deslauriers	9	2011 (± 5.96)	0.20 (± 0.64)	-0.06 (± 0.08)	0.31	0.77	
		Strachan Is	14	NA	-0.06 (± 0.03)	NA	-2.26	0.043*	CT
∑_11_PBDE	Arctic	East Bay	9	NA^a^	0.03 (± 0.02)	NA	1.53	0.17	
		Great Slave Lake	6	2015 (± 2.37)	-0.17 (± 0.18)	0.30 (± 0.19)	-0.98	0.43	
	Atlantic	Gull Is	11	2018 (± 5.24)	-0.05 (± 0.03)	0.04 (± 0.09)	-1.41	0.20	
		Kent Is	10	2021 (± 2.33)	-0.12 (± 0.04)	0.20 (± 0.55)	-3.08	0.022*	IT
	Lake Erie	Middle Is	15	2018 (± 3.73)	0.02 (± 0.03)	-0.05 (± 0.06)	0.79	0.45	
		Port Colborne	14	2019 (± 5.61)	0.05 (± 0.02)	-0.21 (± 0.42)	2.03	0.07	
	Lake Huron	Chantry Is	14	2011 (± 2.27)	0.13 (± 0.16)	-0.05 (± 0.03)	0.81	0.44	
		Double Is	14	2019 (± 12.55)	0.05 (± 0.02)	-0.05 (± 0.38)	2.34	0.042*	LI
	Lake Ontario	Hamilton H	14	2015 (± 2.75)	0.04 (± 0.04)	-0.05 (± 0.03)	0.87	0.40	
		Snake Is	15	NA^a^	-0.03 (± 0.02)	NA	-2.22	0.045*	CT
		Toronto H	13	2014 (± 2.78)	-0.09 (± 0.08)	0.05 (± 0.06)	-1.05	0.32	
	Lake Superior	Agawa Rocks	15	2018 (± 6.84)	0.03 (± 0.04)	-0.05 (± 0.13)	0.68	0.51	
		Granite Is	16	2015 (± 1.04)	0.07 (± 0.02)	-0.06 (± 0.02)	3.43	0.0050*	LI
	Niagara R	Weseloh Rocks	10	2013 (± 6.93)	0.004 (± 0.074)	0.05 (± 0.05)	0.06	0.96	
	St. Lawrence R	Île Bellechasse	11	NA	-0.09 (± 0.03)	NA	-3.50	0.0068*	CT
		Île Deslauriers	10	2022 (± 0.97)	0.02 (± 0.03)	-0.47 (± 0.39)	0.67	0.53	
		Strachan Is	14	NA	-0.05 (± 0.01)	NA	-6.02	0.000060*	CT
BDE-209	Arctic	East Bay	8	2013 (± 2.24)	0.22 (± 0.30)	-0.10 (± 0.05)	0.72	0.50	
		Great Slave Lake	6	2016 (± 0.84)	-0.42 (± 0.09)	0.23 (± 0.09)	-4.81	0.041*	IT
	Atlantic	Gull Is	11	2017 (± 7.99)	-0.02 (± 0.06)	0.04 (± 0.08)	-0.40	0.70	
		Kent Is	10	NA	-0.07 (± 0.03)	NA	-2.43	0.039*	CT
	Lake Erie	Middle Is	15	2015 (± 4.26)	0.06 (± 0.11)	-0.12 (± 0.11)	0.51	0.62	
		Port Colborne	14	2015 (± 1.89)	0.19 (± 0.11)	-0.19 (± 0.11)	1.71	0.12	
	Lake Huron	Chantry Is	14	2016 (± 2.80)	0.04 (± 0.06)	-0.10 (± 0.06)	0.70	0.50	
		Double Is	14	2016 (± 3.50)	0.10 (± 0.07)	-0.06 (± 0.10)	1.45	0.18	
	Lake Ontario	Hamilton H	14	2015 (± 3.58)	0.11 (± 0.10)	-0.07 (± 0.11)	1.14	0.28	
		Snake Is	15	2015 (± 3.12)	0.30 (± 0.17)	-0.06 (± 0.17)	1.76	0.11	
		Toronto H	13	2016 (± 2.12)	0.02 (± 0.07)	-0.22 (± 0.12)	0.23	0.83	
	Lake Superior	Agawa Rocks	15	2015 (± 2.64)	0.08 (± 0.07)	-0.10 (± 0.07)	1.04	0.32	
		Granite Is	16	2014 (± 6.56)	0.09 (± 0.16)	-0.03 (± 0.11)	0.58	0.57	
	Niagara R	Weseloh Rocks	10	2011 (± 1.44)	-0.67 (± 0.56)	0.24 (± 0.11)	1.20	0.28	
	St. Lawrence R	Île Bellechasse	11	NA^a^	-0.11 (± 0.02)	NA	-5.47	0.00040*	CT
		Île Deslauriers	10	2010 (± 0.44)	-0.95 (± 0.26)	0.07 (± 0.03)	-3.59	0.0115*	IT
		Strachan Is	14	NA	0.12 (± 0.04)	NA	3.30	0.0063*	CT
∑DP	Arctic	East Bay	8	2013 (± 1.29)	0.33 (± 0.16)	-0.06 (± 0.05)	2.05	0.10	
		Great Slave Lake	6	2015 (± 2.60)	-0.53 (± 0.46)	0.28 (± 0.13)	-1.14	0.37	
	Atlantic	Gull Is	10	2011 (± 1.61)	0.48 (± 0.44)	-0.15 (± 0.05)	1.09	0.31	
		Kent Is	9	2019 (+ 3.15)	-0.16 (± 0.14)	0.28 (0.34)	-1.21	0.27	
	Lake Erie	Middle Is	15	2014 (± 1.71)	0.53 (± 0.30)	-0.40 (± 0.20)	1.77	0.11	
		Port Colborne	14	2015 (± 3.63)	0.24 (± 0.20)	-0.12 (± 0.21)	1.20	0.26	
	Lake Huron	Chantry Is	14	2011 (± 2.62)	0.17 (± 0.29)	-0.08 (± 0.03)	0.60	0.57	
		Double Is	14	2014 (± 1.19)	0.33 (± 0.14)	-0.21 (± 0.07)	2.37	0.039*	EI
	Lake Ontario	Hamilton H	14	NA^a^	-0.10 (± 0.04)	NA	-2.41	0.033*	CT
		Snake Is	15	2015 (± 2.26)	0.39 (± 0.28)	-0.28 (± 0.19)	1.41	0.19	
		Toronto H	13	2012 (± 1.32)	0.23 (± 0.16)	-0.12 (± 0.04)	1.42	0.19	
	Lake Superior	Agawa Rocks	15	2015 (± 2.28)	0.09 (± 0.11)	-0.18 (± 0.08)	0.82	0.43	
		Granite Is	16	NA	-0.08 (± 0.03)	NA	-2.50	0.026*	CT
	Niagara R	Weseloh Rocks	10	2010 (± 1.23)	0.43 (± 0.35)	-0.09 (± 0.07)	1.25	0.26	
	St. Lawrence R	Île Bellechasse	11	2013 (± 1.36)	0.37 (± 0.17)	-0.29 (± 0.10)	2.19	0.06	
		Île Deslauriers	10	2017 (± 1.82)	0.13 (± 0.09)	-0.25 (± 0.10)	1.40	0.21	
		Strachan Is	14	2019 (± 1.99)	-0.07 (± 0.05)	0.29 (± 0.19)	1.33	0.21	

^a^ indicates that breakpoint analysis could not be completed; linear regression result presented

Significant models are denoted by an asterisk (*) next to the Pr( >|t|) value and are assigned a trend model. Trend model abbreviations are as follows: increasing trend (IT), listing improvement (LI), nomination improvement (NI), early improvement (EI), or consistent trend (CT)

### Sum 11 PBDEs

**Fig. 3:**
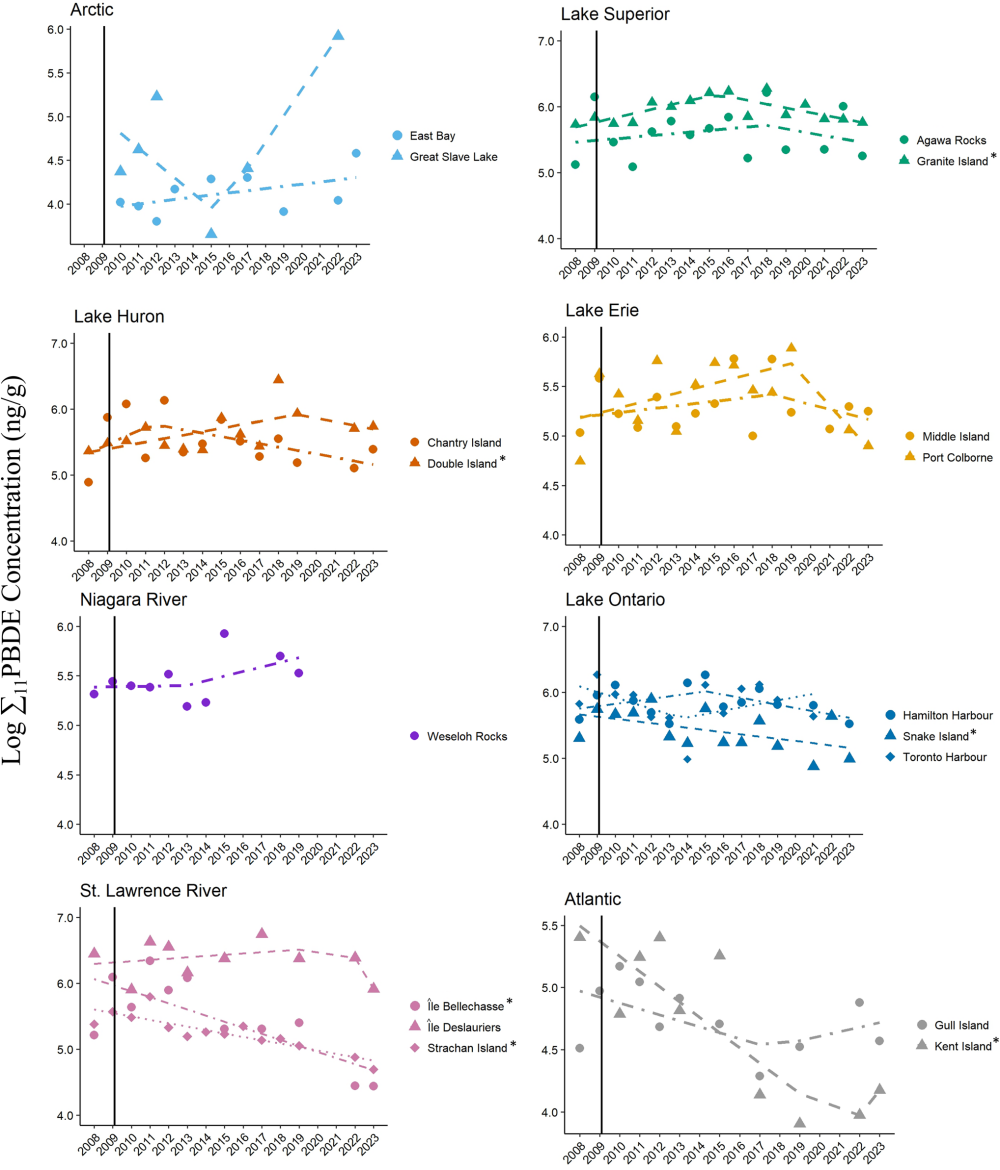
Trends and breakpoints for concentrations (ln ng/g, wet weight) of ∑_11_PBDE corresponding to the penta- and octa-commercial mixtures in herring gull eggs from 17 colonies in Canada from 2008–2023. The year of listing of penta- and octa-BDE mixtures to Annex A of the Stockholm Convention (2009) is shown as a solid vertical line. Models that are statistically significant are denoted by an asterisk (*) beside the colony name

Significant temporal changes in Σ_11_PBDE concentrations were also evident in eggs from six herring gull colonies (Fig. 3; Table 1). Eggs from two colonies, Double Island on Lake Huron and Granite Island on Lake Superior, followed the listing improvement (LI) model with annual increases of 5% and 7% (exp(0.05[β]) ng/g/year and exp(0.07[β]) ng/g/year, respectively), significant breakpoints (2019 and 2015), followed by annual decreases of 5% and 6% (exp(-0.05[β]) ng/g/year and exp(-0.06[β]) ng/g/year, respectively; t > 2.34, *p* < 0.05). Breakpoint years for eggs from these two colonies were at least six years following listing of penta-BDE and octa-BDE commercial mixtures in 2009. Eggs from Kent Island followed the increasing trend (IT) model with initially an annual decrease of 11% (exp(-0.12[β]) ng/g/year), significant breakpoint at 2021 (12 years following the year of listing (2009)), and then an annual increase of 22% in Σ_11_PBDE concentrations (exp(0.20[β]) ng/g/year; t = -3.08, *p* < 0.03). Significant decreases in egg Σ_11_PBDE concentrations supported the CT model that was found at three colonies at Snake Island on Lake Ontario and two colonies on the St. Lawrence River at Île Bellechasse and Strachan Island over the study period (annual decreases ranged from 9 to 3% or exp(-0.09) – (exp(-0.03[β]) ng/g/year; t < − 2.22, *p* < 0.05).

### BDE-209

**Fig. 4:**
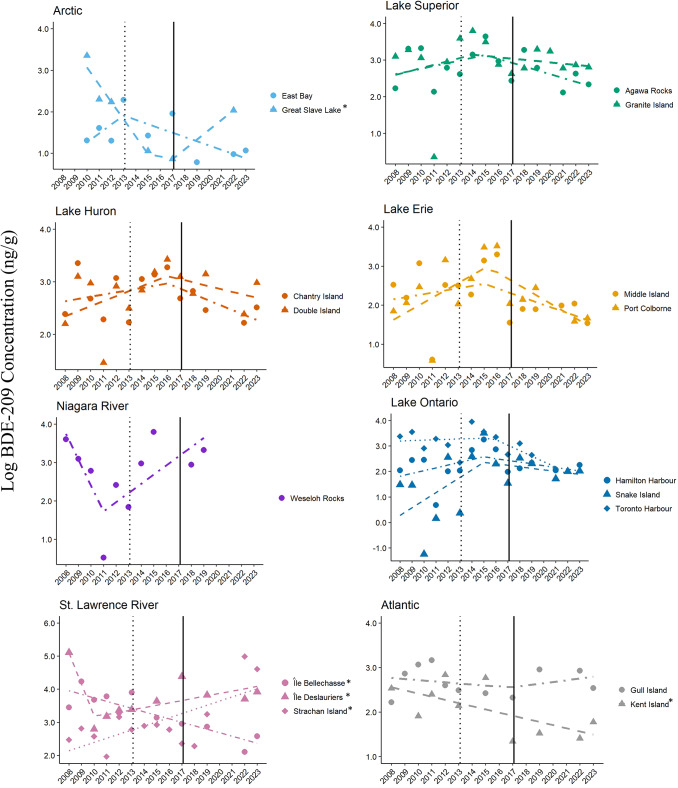
Trends and breakpoints for concentrations (ln ng/g, wet weight) of BDE-209 in herring gull eggs from 17 colonies in Canada from 2008–2023. The year of nomination of deca-BDE to Annex A of the Stockholm Convention (2013) is shown as a dotted vertical line and the year of listing (2017) is shown as a solid vertical line. Models that were statistically significant are denoted by an asterisk (*) beside the colony name

Significant temporal changes in BDE-209 concentrations occurred in eggs from five colonies (Fig. 4; Table 1). Eggs from Great Slave Lake followed the IT model with initially an annual decrease of 34% (exp(− 0.42[β]) ng/g/year), significant breakpoint at 2016 (three years following nomination (2013) and one year prior to listing (2017)), and then an annual increase of 26% in BDE-209 concentrations (exp(0.23[β]) ng/g/year; t = − 4.81, *p* < 0.05). Eggs from Île Deslauriers also followed the IT model with initially an annual decrease of 61% (exp(− 0.95[β]) ng/g/year), significant breakpoint at 2010 (three years prior to nomination (2013)), and then an annual increase of 7% in BDE-209 concentrations (exp(0.07[β]) ng/g/year; t = − 3.59, *p* < 0.02). Egg BDE-209 concentrations at two colonies, Kent Island and Île Bellechasse, followed the CT model and declined significantly (7% annual decrease or exp(-0.07[β]) ng/g/year and 10% annual decrease or exp(-0.11[β]) ng/g/year, respectively; t < − 2.43, *p* < 0.05). In contrast, BDE-209 concentrations in eggs from Strachan Island followed the CT model but increased significantly over the study period (13% annual increase or exp(0.12[β]) ng/g/year; t = 3.30, *p* = 0.006).

### Dechlorane Plus

**Fig. 5:**
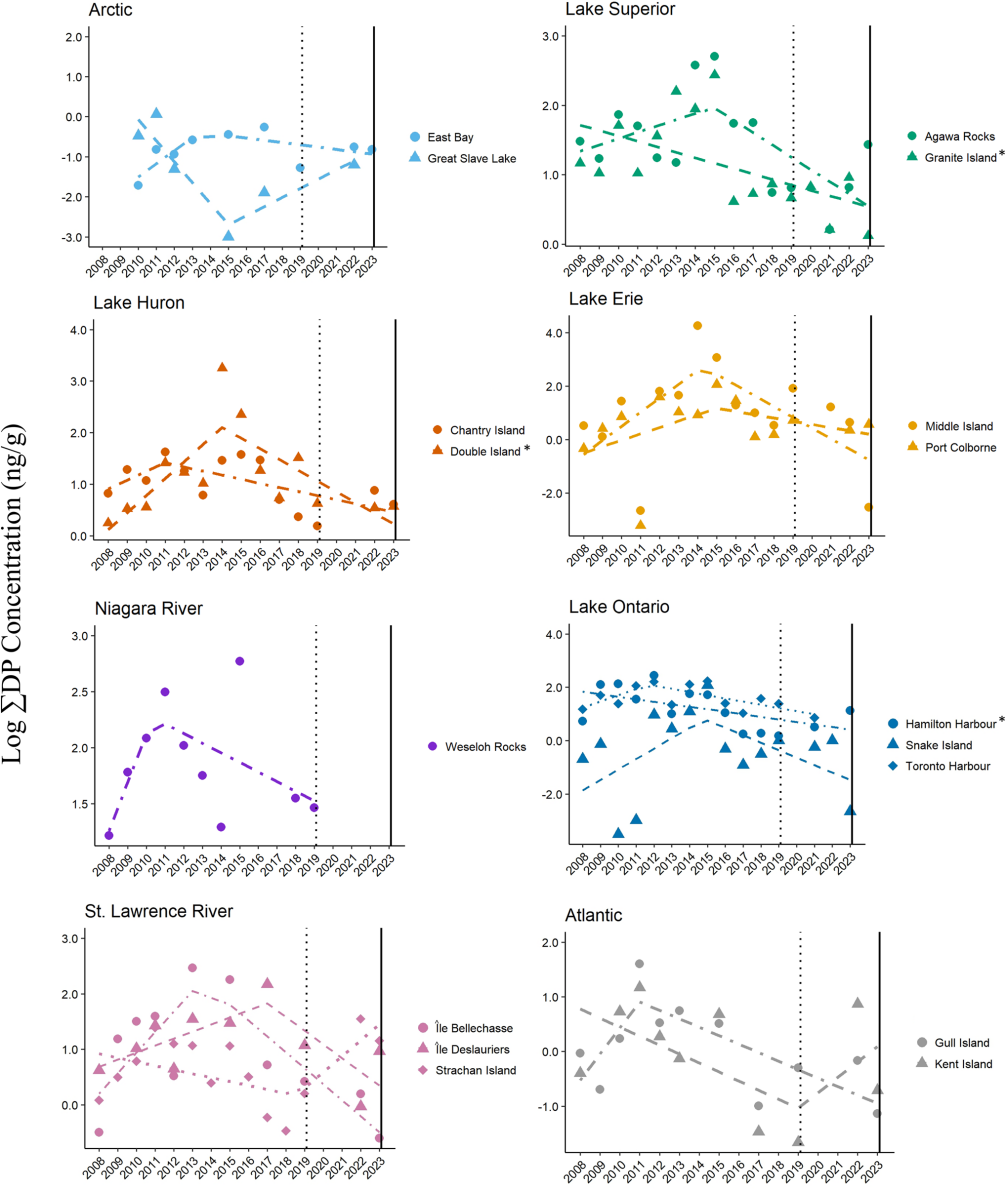
Trends and breakpoints for concentrations (ln ng/g, wet weight) of Dechlorane Plus (∑DP) in herring gull eggs from 17 colonies in Canada from 2008–2023. The year of nomination for DP to Annex A of the Stockholm Convention (2019) is shown as a dotted vertical line and the year of listing (2023) is shown as a solid vertical line. Models that were statistically significant are denoted by an asterisk (*) beside the colony name

Significant temporal changes in ∑DP concentrations were found in herring gull eggs at three colonies (Fig. 5; Table 1). Eggs from Double Island followed the EI model with initially an annual increase of 39% (exp(0.33[β]) ng/g/year), significant breakpoint in 2014 (five years prior to nomination), followed by an annual decrease of 19% (exp(-0.21[β]) ng/g/year; t = 2.37, *p* < 0.04). Two colonies, Hamilton Harbour on Lake Ontario and Granite Island on Lake Superior, supported the CT model showing significant declines in egg ∑DP concentrations of 10% and 8% annually, respectively (exp(− 0.10[β]) ng/g/year and exp(-0.08[β]) ng/g/year, respectively; t < − 2.41, *p* < 0.04).

### Spatial Comparisons

Based on likelihood‐ratio tests comparing full models vs null models (with or without Region as a factor), the addition of Region to the full models improved prediction for all HFRs (Table S6). Therefore, we included Region in all models. Concentrations of all four HFRs differed among regions (Σ_11_PBDE [F_[3,12.20]_ = 19.60,  *p*< 0.0001), BDE-209 [F_[3, 11.69]_ = 13.47,  *p*< 0.0001], HBCDD [F_[3,11.71]_ = 7.28,  *p*= 0.0051] and ∑DP [F_[3, 9.98]_ = 26.01, *p*=0.0001; Supplementary Information, Figures S1a,b,c,d). Mean Σ_11_PBDE (Supplementary Information, Tables S7a, S7b) and BDE-209 (Supplementary Information, Tables S8a, S8b) differed in all four regions compared to the overall mean, with concentrations highest in eggs from the St. Lawrence River and Great Lakes. Mean HBCDD concentrations (Supplementary Information, Tables S9a, S9b) in eggs were higher from the St Lawrence River and Great Lakes compared to the overall mean, whereas for ∑DP, mean concentrations (Supplementary Information, Tables S10a, S10b) in eggs were higher from the St. Lawrence River and Great Lakes and lower from the Arctic compared to the overall mean.

Based upon the LMM, Colony (nested within Region) predicted between 8.8 and 18.4% of the variance in HFR concentrations for HBCDD – Σ_11_PBDE, respectively; hence Colony (nested within Region) was about equally important for each HFR regardless of Region (Supplementary Information, Tables S7a, S8a, S9a, S10a). However, the variance explained by Year varied from 4.2, 7.0 and 11.7% for BDE-209, Σ_11_PBDE and ∑DP, respectively, but as high as 25.2% for HBCDD (Supplementary Information, Tables S7a, S8a, S9a, S10a).

## Discussion

Chemicals such as HFRs have been listed under the SC-POPs in an effort to reduce levels of harmful persistent chemicals in the environment, and the exposure of wildlife and humans to these chemicals. We hypothesized that concentrations of HFRs in herring gull eggs would decline in response to their listing or nomination to the SC-POPs. However, despite more than a decade of regulations for some of these compounds, the present study found little evidence that these global regulations alone have been effective in reducing HFR concentrations in herring gull eggs in Canada. For the large majority of comparisons (75%, 51/68), temporal trends for HFRs in herring gull eggs were stable. Some significant trends were found, but the timing of these temporal changes was inconsistent across colonies and was rarely associated with Stockholm Convention processes indicating that other factors contributed more to observed declines at colonies (see below).

Focusing on the few (three) colonies that showed a breakpoint when the chemical was nominated or listed under the SC-POPs and then subsequently declined in concentration, these varied by compound, by colony, and by waterbody. Changes in egg HBCDD concentrations followed a nomination-improvement model at one colony (Gull Island [Atlantic]), but were otherwise stable at all but two colonies (Strachan Island [St. Lawrence River] and Kent Island [Atlantic]) where HBCDD followed a consistent declining trend. For two colonies (Double Island [Lake Huron] and Granite Island [Lake Superior]), changes in egg Σ_11_PBDE concentrations followed a listing-improvement model, whereas Σ_11_PBDEs had consistently declining trends at three colonies and were stable at 11 colonies. Not only did these three breakpoint examples differ by compound, but all colonies were in different bodies of water (Atlantic Ocean, and lakes Huron and Superior), further indicating a lack of consistency in these breakpoints. At one additional colony (Double Island [Lake Huron]), changes in ∑DP concentrations followed an early improvement model that was five years prior to the year of nomination, a trend that was not reported elsewhere or for any other HFR. At the remaining colonies, egg ∑DP concentrations showed consistently declining trends (2) or were stable (14).

A portion of comparisons (13%, 9/68) indicated consistent and significant declines in HFR concentrations at colonies since monitoring was initiated in 2008. These declines also coincided with years of nomination for HBCDD (2008) and listing for PBDEs (2009) but were several years earlier than nomination years for BDE-209 and ∑DP. Σ_11_PBDEs had the most significant declines (3/17) at herring gull colonies, followed by HBCDD, BDE-209, and ∑DP (2/17 each). Conversely, temporal trends for some HFRs at some colonies were unexpected: one colony showed a temporal increase for BDE-209 (Strachan Island [St. Lawrence River]) and three colonies, after a period of decreasing trends, showed increasing trends in concentrations of Σ_11_PBDEs (Kent Island [Atlantic]) and BDE-209 (Great Slave Lake [Arctic], Île Deslauriers [St. Lawrence River]). Temporal trends in egg HFR concentrations were also not associated with any particular waterbody, with the number of significant trends ranging from zero at colonies on Lake Erie and the Niagara River to five at the three colonies on the St. Lawrence River. Overall, temporal trends for these compounds did not follow patterns of expected declines in concentrations and were not consistent within colonies or waterbodies.

Earlier studies of temporal trends of HFRs in eggs of herring gulls – from several of the same Great Lakes colonies in this study–indicate that PBDE concentrations associated with each of the penta- and octa BDE commercial mixtures increased from the early 1980s, reached a peak in 2000, and then declined post-2000 (Norstrom et al. 2002; Gauthier et al. 2008; Su et al. 2015). These trends also coincided with the voluntary phase-out in production by manufacturers and regulatory processes by governments of the two BDE commercial mixtures in Canada and the United States in the early-to mid-2000s (Environment Canada 2011; USEPA, 2014). Similar temporal patterns were also evident in eggs of other bird species on the west coast and Arctic regions of Canada (Miller et al. 2014; Braune et al. 2015). Thus, federal efforts to regulate these two BDE mixtures and initiated several years prior to listing under the Stockholm Convention in 2009 were likely more important contributors to observed trends in herring gull eggs post-2000. Previous studies suggested that enactment of federal regulations are more influential on concentrations and trends of POPs in the environment than is the time of listing under the Stockholm Convention (Hung et al. 2016; Wöhrnschimmel et al. 2016). Federal regulations are one of the main instruments through which the Government of Canada strives to meet its obligations under the SC-POPs; therefore, international and federal actions under the SC-POPs are linked and parsing out the relative contributions of each is challenging. While the monitoring period in this study does not capture the year when concentrations of these compounds were highest in gull eggs, i.e., early 2000s, Σ_11_PBDEs concentrations showed significant declines at some colonies more than ten years after federal and global regulations were implemented and suggest that these regulatory measures continue to be effective.

In contrast to temporal declines reported for Σ_11_PBDEs in bird eggs in Canada after 2000, concentrations of HBCDD, BDE-209, and ΣDP in herring gull eggs from Great Lakes colonies showed steady increases from the mid-1990s to mid-2010s (Gauthier et al. 2008; Gauthier and Letcher 2009; Su et al. 2015). In this current assessment, significant declines in egg concentrations and the downward trajectory for some HFRs following a significant breakpoint at some gull colonies across Canada are encouraging. Again, apportioning trends to the timing of specific regulations is challenging since federal regulations were also in effect for two HFRs: voluntary phase-outs of exports of commercial deca-BDE by the major USA manufacturers to Canada were completed in 2013 and Canadian regulations of HBCDD came into force in 2016 (Abbasi et al. 2015; Government of Canada 2016). In addition to the actions of different regulatory agencies, the roll-out of regulatory actions can take several years. Under the Stockholm Convention, for instance, regulations are phased in over a few years between nomination and formal listing. This signals industry of likely upcoming regulation that results in a gradual phasing out of the chemical. Further, POPs may be listed to Annex A of the Stockholm Convention for elimination with time-limited specific exemptions and Parties to the Convention may register for these exemptions to be able to continue production and use. For DP, Canada does not currently prohibit the manufacture, use, sale or import and/or of products containing DP (Government of Canada, 2022). In the absence of federal regulations, egg ∑DP concentrations showed significant declines at three colonies well before years of nomination and listing of DP under the SC-POPs (Granite Island, Double Island, and Hamilton Harbour; Fig. 5). This suggests that impending regulatory restrictions under the Stockholm Convention may have been effective. Currently, Canada is proposing further restrictions on products containing HBCDD and PBDEs (including deca-BDE) as well as restrictions on DP outside of the commitments of the Stockholm Convention (Government of Canada, 2022). As part of ECCC’s Chemicals Management Plan, ongoing years of herring gull egg monitoring across Canada will continue to examine how these additional actions reduce HFR exposure in gulls as well as in other environmental matrices.

There have been cases where changes in regulations have resulted in reductions in environmental levels soon after. For example, in 1974, PCB production in North America became restricted from “open systems”, to reduce the potential of PCBs leaching into the environment; PCB concentrations in Great Lakes gulls rapidly dropped from 1974 onward (de Solla et al. 2016). Similarly, PFOS concentrations in double-crested cormorants and rhinoceros auklets (*Cerorhinca monocerata*) along the Canadian Pacific coast started to decline around 2000, when 3M announced their phasing out of PFOS production (Kesic et al. 2023). However, once regulations are enacted, their environmental effects may not be immediate, further adding to the complexity of associating timing and effectiveness of regulations with predicted reductions of POPs in the environment. Following the cessation of manufacturing of products containing the chemical, emissions may continue to occur throughout the lifecycle and disposal phases of the product causing a long lag time before emissions dissipate (UNEP/AMAP, 2011). Long-term reservoirs can accumulate in soil, sediments, and surface waters, which subsequently turn into secondary sources to the environment through remobilization of POPs. For example, following years of consistent reductions of polychlorinated naphthalenes (PCNs) in both herring gull eggs and lake trout (*Salvelinus namaycush*), sediment dredging in the Detroit River caused a release of PCNs into Lake Erie, causing a resurgence of concentrations in both species which lasted for 5 to 10 years (McGoldrick et al. 2018). Despite bans on the production of polychlorinated biphenyls (PCB) in the 1970s, following changes in water–air exchange fluxes, PCB concentrations in air would sometimes increase over a period of years, as fluxes of PCBs switched from net areal deposition in surface waters to net volatilization into the atmosphere (Zhao et al. 2015). Releases from both in-use and discarded HFR-containing products may also continue for years. Disposal of these products to landfills are other important sources of HFRs to the environment including to nesting birds feeding nearby (Chen et al. 2013). Atmospheric transport and deposition of HFRs originating from urban and industrial sources to remote sites could also result in a lag period associated with the timing of the effects of regulations (Miller et al. 2014). We speculate that, post-regulation, these factors could result in ongoing releases to the environment and that delay and contribute to differences in temporal patterns of egg HFRs reported spatially across gull colonies and waterbodies.

Biodegradation of PBDEs also influence temporal patterns reported in gulls following regulation. Dosing studies of birds with penta-BDE, octa-BDE, and deca-BDE commercial mixtures suggest that PBDE congeners can be biotransformed to lower brominated BDE congeners in the embryo and tissues (Van den Steen et al*.*, 2007; McKernan et al. 2010). PBDE debromination can also occur in abiotic matrices via phytochemical and microbial pathways in the environment (Rodenburg et al. 2014). Due to its high potential to bind to particles, significant reserves of BDE-209 can accumulate in sediment where it breaks down to lower brominated BDE congeners such as those associated with penta- and octa-BDE commercial mixtures (Ross et al. 2009; Orihel et al. 2016). While the debromination process for BDE-209 is likely very slow, it can provide a long-term source for lower brominated BDE congeners that are more bioavailable in the aquatic foodweb (Orihel et al. 2016).

As predicted, HFR concentrations were generally higher in gull eggs from the Great Lakes – St. Lawrence River basin, and lower in eggs from the Arctic and Atlantic regions, compared to the overall average. For remote locations, such as the Arctic, the main source of POPs is generallygenerally considered to be long distance transport, either by long-range atmosphere transport or by long-range ocean transport (Li et al. 2023), depending on the volatility of the chemical. Consequently, concentrations of POPs tend to be lower in biota from the Arctic compared to comparable biota from regions with significant local sources. The trend towards higher HFRs in the Great Lakes and St. Lawrence River basin compared to marine sites has been previously reported (Chen et al. 2012), which is consistent with sources from localized large population centres and significant industry. That there are many potential factors affecting body or egg burdens of HFRs may be why the variance that both Colony and Year explained was not higher. Colony location explained between 8.8 and 18.4%, which indicates a relatively low variability in responses in HFR among colonies. Given that all these HFRs are lipophilic, and are sourced by diet, such high similarity among Colonies is not that surprising. The variation explained by Year, however, ranged between 4.2 – 25.2%; with HBCDD having a large temporal contribution. Regulations and production histories were unique to each compound, which may explain the larger difference explained by time among HFRs

Several ecological considerations may affect temporal changes of HFRs in biota. For example, changes in food web dynamics both in freshwater ecosystems like the Great Lakes and in marine environments may affect the trophic position of gulls (Hebert and Weseloh 2006). Herring gulls are facultative piscivores, as they are flexible generalist omnivores that will deviate from a piscivorous diet in response to low fish populations. Generally, alterations in foraging of gulls towards greater piscivorous diets were associated with greater egg burdens of PCBs (Hebert et al. 2000), whereas increases in terrestrial foraging were associated with higher burdens of UV stabilizers in eggs (Lu et al. 2018). Although herring gulls integrate contaminant exposure over relatively small spatial scales surrounding nesting colonies, such that egg contaminant burdens primarily reflect local foraging environments, some colonies, especially in the Great Lakes, may be within foraging range of landfills, which may contribute to HFR burdens in gull eggs.

While herring gulls migrate both within and occasionally outside the Laurentian Great Lakes and overwintering behavior has been shown to influence egg POP burdens (Hebert 1998; Leat et al. 2013), it is possible that endogenous lipid reserves formed during overwintering or migration may influence egg burdens at the breeding grounds. However, while migration and overwintering distributions have some influence on chemical concentrations in herring gull eggs, approximately 80% of PCB burdens in gull eggs in colonies in the Great Lakes were predictable by breeding colony (Hammond et al. 2025), and hence concentrations are more substantially influenced by dietary exposure at their breeding grounds (Leat et al. 2013; Miller et al. 2020). Additionally, egg formation strategy across herring gull colonies and subpopulations may not be uniform. One study from Finland found that female herring gulls lose more weight during egg formation and laying than they gain after arriving at the breeding colony, indicating that at least some endogenous lipid is used for egg formation (Hario et al. 1991). However, a study of herring gulls at Great Slave Lake found that little or no endogenous reserves were used for reproduction (Hobson et al. 2000). We were unable to quantify the importance of these factors in determining egg burdens, although generally site locations of breeding colonies are the main determinant of egg burdens (Hebert 1998), and we found that region was a significant predictor of HFR burdens, which also suggests that HFRs in herring gull eggs reflect breeding location. Nonetheless, using avian eggs for monitoring has proved to be a reliable and low impact method for monitoring chemicals in the environment (Norstrom et al. 2002; Su et al. 2015; Hammond et al 2025).

## Conclusions

Overall, the results of our study suggest that nomination or listing of target HFRs under the Stockholm Convention largely did not correspond temporally with reductions in HFR concentrations in herring gull eggs across Canada. Other factors were likely greater contributors to the temporal trends of HFRs in gull eggs. Federal regulations restricting production, usage, and/or trade, which are often enacted independent of the Stockholm Convention, may be more important in driving emissions of HFRs and other substances. Given the long environmental half-lives of these chemicals, a longer time frame after changes in regulation may be required for significant changes to be detected. Proximity to urban sources, production and use volumes, continued emissions from products and waste containing these flame retardants, environmental reservoirs, and biological factors influencing herring gull exposure, feeding ecology, and biomagnification likely contribute to the trends in HFRs we observed in Canadian gull eggs. Given the substantial annual variation in chemical concentrations at each colony, many years of monitoring is required to determine the success, or failure, of regulation to reduce environmental levels of persistent chemicals.

## Supplementary Information

Below is the link to the electronic supplementary material.

Supplementary Material
