# Supplementary material for: Effect of Stockholm Convention Listing on Temporal Trends of Halogenated Flame Retardants in Herring Gull Eggs in Canada (2008–2023)
Source: Arch Environ Contam Toxicol. 2026 Feb 2;90(2):11. doi: 10.1007/s00244-025-01173-2 (PMC12864295; doi:10.1007/s00244-025-01173-2)
Supplement: Supplementary file 1 — Supplementary Material [file 244_2025_1173_MOESM1_ESM.docx]

**Supplementary Information**

**Effect of Stockholm Convention Listing on Temporal Trends of Halogenated Flame Retardants in Herring Gull Eggs in Canada (2008–2023)**

Vanderlip, H.L.^1^, Hughes, K.D.^2^ ([0009-0009-8306-0618](https://orcid.org/0009-0009-8306-0618)), Orihel, D.M.^1,3^ (0000-0002-6933-3650), Friesen, V.L.^1,3^ ([0000-0002-4921-1170](https://orcid.org/0000-0002-4921-1170)), de Solla, S.R.^4^ ([0000-0002-8491-4285](https://orcid.org/0000-0002-8491-4285)), Letcher, R.J. ^5^ ([0000-0002-8232-8565](https://orcid.org/0000-0002-8232-8565)), Martin, P.A.^4^, Lavoie, R.A.^6^ ([0000-0003-3381-3254](https://orcid.org/0000-0003-3381-3254)), Eng, M.L.^7^ ([0000-0002-4217-619X](https://orcid.org/0000-0002-4217-619X)), Provencher, J.F.^5^ ([0000-0002-4972-2034](https://orcid.org/0000-0002-4972-2034))

^1^ Department of Biology, Queen’s University, Kingston, Ontario, Canada

^2^ Broadwing Biological Consulting, Port Perry, Ontario, Canada

^3^ School of Environmental Studies, Queen’s University, Kingston, Ontario, Canada

^4^ Ecotoxicology and Wildlife Health Division, Science and Technology Branch, Environment and Climate Change Canada, Burlington, Ontario, Canada

^5^ Ecotoxicology and Wildlife Health Division, Science and Technology Branch, Environment and Climate Change Canada, Ottawa, Ontario, Canada

^6^ Ecotoxicology and Wildlife Health Division, Science and Technology Branch, Environment and Climate Change Canada, Québec, Québec, Canada

^7^ Ecotoxicology and Wildlife Health Division, Science and Technology Branch, Environment and Climate Change Canada, Dartmouth, Nova Scotia, Canada

Corresponding author: Shane de Solla (shane.desolla@ec.gc.ca)

Table S1. Colony locations and years of HFR chemical analysis in egg collections of herring gulls (*Larus argentatus = Larus smithsonianus*) from 2008–2023.

| **Region** | **Colony** | **Province/State** | **Latitude** | **Longitude** | **Years of HFR Analysis** | **No. Years Assessed** |
| --- | --- | --- | --- | --- | --- | --- |
| Arctic | East Bay | NU | 64.0297 | -81.7880 | 2010–2023 | 9 |
|  | Great Slave Lake | NT | 62.6016 | -115.5263 | 2010–2022 | 6 |
| Atlantic | Gull Is. | NL | 47.2586 | -52.7728 | 2008–2023 | 11 |
|  | Kent Is. | NB | 44.5833 | -66.7540 | 2008–2023 | 10 |
| Lake Erie | Middle Is. | ON | 41.6817 | -82.6817 | 2008–2023 | 15 |
|  | Port Colborne | ON | 42.8683 | -79.2583 | 2008–2023 | 14 |
| Lake Huron | Chantry Is. | ON | 44.4928 | -81.4033 | 2008–2023 | 14 |
|  | Double Is. | ON | 46.1734 | -82.8642 | 2008–2023 | 14 |
| Lake Ontario | Hamilton Harbour | ON | 43.3070 | -79.8048 | 2008–2023 | 14 |
|  | Snake Is. | ON | 44.1908 | -76.5431 | 2008–2023 | 15 |
|  | Toronto Harbour | ON | 43.6214 | -79.3313 | 2008–2021 | 13 |
| Lake Superior | Agawa Rocks | ON | 47.3508 | -84.7007 | 2008–2023 | 15 |
|  | Granite Is. | ON | 48.7204 | -88.4602 | 2008–2023 | 16 |
| Niagara River | Weseloh Rocks | ON | 43.0754 | -79.0701 | 2008–2015 | 8 |
|  | Buffalo Harbor | NY | 42.8843 | -78.9009 | 2018 & 2019 | 2 |
| St. Lawrence River | Île Bellechasse | QC | 46.9314 | -70.7672 | 2008–2023 | 11 |
|  | Île Deslauriers | QC | 45.7122 | -73.4408 | 2008–2023 | 10 |
|  | Strachan Is. | ON | 45.0211 | -74.8115 | 2008–2023 | 14 |

**Table S2.** Method limit of detection (MLOD; ng/g wet weight), method limit of quantitation (MLOQ; ng/g wet weight), and percent of samples with reportable HFR concentrations, i.e., above MLODs or MLOQs, shown in brackets for 11 BDE congeners, BDE-209, HBCDD, and the two isomers of DP, *anti-*DP and *syn-*DP, in egg samples of herring gulls from 17 colonies from 2008–2023. MLODs and MLOQs are shown as means for 11 BDE congeners quantified since MLODs and MLOQs were congener specific for analyses conducted between 2014–2023. For brevity, these are grouped together for years 2016–2022 since these did not change over the 7 years of chemical analysis. NA indicates that MLOQs are not available.

| **Year** | **2008** | **2009** | **2010** | **2011** | **2012** | **2013** | **2014** | **2015** | **2016–2022** | **2023** |
| --- | --- | --- | --- | --- | --- | --- | --- | --- | --- | --- |
| **No. Egg Samples** | **31** | **19** | **49** | **49** | **49** | **43** | **11** | **49** | **182** | **29** |
|  | MLOD/  MLOQ | MLOD/  MLOQ | MLOD/  MLOQ | MLOD/  MLOQ | MLOD/  MLOQ | MLOD/  MLOQ | MLOD/  MLOQ | MLOD/  MLOQ | MLOD/  MLOQ | MLOD/  MLOQ |
| 11 BDE Congeners | 0.1/NA  (95%) | 0.1/NA  (98%) | 0.1/NA (96%) | 0.1/NA (97%) | 0.1/NA (94%) | 0.1/NA (95%) | 0.23/0.75  (73%) | 0.06/0.17  (81%) | 0.05/0.15  (89%) | 0.06/0.20 (91%) |
| BDE-209 | 0.03/0.11 (97%) | 0.1/NA  (100%) | 1.0/NA  (98%) | 5.00/NA  (84%) | 5.00/NA  (94%) | 5.00/NA (79%) | 0.65/2.2  (100%) | 0.47/1.4  (94%) | 0.47/1.4  (97%) | 0.05/0.17  (100%) |
| HBCDD | 0.28/1.0  (100%) | 0.1/NA (58%) | 1.0/NA (100%) | 1.0/NA (94%) | 1.0/NA (73%) | 1.0/NA  (93%) | 0.49/1.63  (100%) | 0.03/0.10 (98%) | 0.03/0.10  (98%) | 0.19/0.62  (100%) |
| *anti*-DP | 0.01/0.03 (97%) | 0.1/NA  (100%) | 0.1/NA  (90%) | 0.1/NA  (84%) | 0.1/NA  (98%) | 0.1/NA  (98%) | 0.23/0.77  (100%) | 0.04/0.13 (84%) | 0.10/0.29 (94%) | 0.06/0.21 (86%) |
| *syn*-DP | 0.03/0.12 (94%) | 0.1/NA  (95%) | 0.1/NA  (80%) | 0.1/NA  (61%) | 0.1/NA  (82%) | 0.1/NA  (91%) | 0.18/0.60 (64%) | 0.05/0.15  (82%) | 0.05/0.15 (66%) | 0.06/0.20 (62%) |

**Contribution of lipids to models predicting HFR concentrations.**

We found a lack of correlations between contaminants and lipid content. However, to further determine if lipids are an important predictor of flame retardant concentrations in gull eggs that should be added to our models, we conducted a formal test of the full models, including lipids in predicting HFR (HBCDD, ∑_11_PBDE, BDE-209 or ∑DP) concentrations with the reduced models, lacking lipids.
The full model is:

$$y_{ijk}=\beta_{0}+\beta_{Region(i)}+\beta_{Lipid}\cdot{Lipid}_{ijk}+u_{ij}+v_{k}+\epsilon_{ijk}$$

And the reduced model, without lipids, is:

$$y_{ijk}=\beta_{0}+\beta_{Region(i)}+u_{ij}+v_{k}+\epsilon_{ijk}$$

where,

$y_{ijk}$= log-transformed HFR concentrations for Colony *j* in Region *i* during Year *k*$\beta_{0}$= overall intercept
$\beta_{\text{Region}(i)}$= fixed effect of Region *i*$\beta_{\text{Lipid}}$= fixed effect of lipid percentage
$u_{ij}$= random intercept for Colony *j* nested within Region *i*$v_{k}$= random intercept for Year *k*$\varepsilon_{ijk}$= residual error

**Table S3**. Log-likelihood tests of full (with lipid) and reduce models (without lipids) to Linear Mixed Models predicting HFR concentrations in gull eggs, presented in main text.

|  | Model | npar | AIC | BIC | logLik | deviance | χ2 | Df | Pr(> χ2) |
| --- | --- | --- | --- | --- | --- | --- | --- | --- | --- |
|  | Reduced | 7 | 945.6 | 975.24 | -465.79 | 931.59 |  |  |  |
| ∑_11_PBDEs | Full | 8 | 947.4 | 981.31 | -465.71 | 931.42 | 0.168 | 1 | 0.6815 |
|  | Reduced | 7 | 1336.2 | 1365.8 | -661.07 | 1322.2 |  |  |  |
| BDE209 | Full | 8 | 1338.1 | 1372 | -661.07 | 1322.1 | 0.009 | 1 | 0.9227 |
|  | Reduced | 7 | 1566.6 | 1596 | -776.31 | 1552.6 |  |  |  |
| HBCDD | Full | 8 | 1568.5 | 1602.1 | -776.24 | 1552.5 | 0.151 | 1 | 0.6979 |
|  | Reduced | 7 | 1572.5 | 1602.2 | -779.28 | 1558.5 |  |  |  |
| Sum DP | Full | 8 | 1573.5 | 1607.4 | -778.76 | 1557.5 | 1.024 | 1 | 0.3116 |

Npar: number of parameters; AIC: Akaike Information Criterion; BIC; Bayesian Information Criterion; logL: log-likelihood

The addition of lipids did not improve any of the models, for HBCDD, ∑_11_PBDE congeners, BDE-209 or ∑DP, and hence we excluded lipids from the models in the main text for assessing temporal or spatial trends.

**Table S4**. Mean (+SE) egg volume (cm^3^), egg mass (g) and percent lipid in herring gull eggs at each study colony. Means for egg volume and mass are based on egg collections from 2008–2019 and means for percent lipid are based on collections from 2008–2023 (where data are available).

| **Region** | **Colony** | **Province** | **Mean Egg Volume (cm^3^)** | **Mean Egg Mass (g)** | **Mean Lipid (%)** |
| --- | --- | --- | --- | --- | --- |
| Arctic | East Bay | NU | 90.38 (+1.04) | 87.65 (+3.21) | 7.78 (+0.42) |
|  | Great Slave Lake | NT | 86.93 (+1.39) | 80.13 (+2.47) | 7.63 (+0.31) |
| Atlantic | Gull Is. | NL | 85.89 (+1.19) | 85.90 (+1.20) | 7.88 (+0.20) |
|  | Kent Is. | NB | 82.52 (+0.35) | 80.45 (+1.58) | 8.04 (+0.22) |
| Lake Erie | Middle Is. | ON | 87.04 (+0.55) | 94.14 (+1.07) | 8.03 (+0.17) |
|  | Port Colborne | ON | 87.29 (+0.74) | 92.99 (+0.82) | 7.91 (+0.28) |
| Lake Huron | Chantry Is. | ON | 86.08 (+0.67) | 91.69 (+1.99) | 7.82 (+0.22) |
|  | Double Is. | ON | 81.08 (+0.90) | 83.53 (+1.02) | 8.12 (+0.19) |
| Lake Ontario | Hamilton Harbour | ON | 86.67 (+0.58) | 91.60 (+1.61) | 8.10 (+0.21) |
|  | Snake Is. | ON | 89.94 (+0.57) | 93.36 (+1.51) | 7.85 (+0.25) |
|  | Toronto Harbour | ON | 84.31 (+1.03) | 87.87 (+1.48) | 8.35 (+0.27) |
| Lake Superior | Agawa Rocks | ON | 81.74 (+0.62) | 84.60 (+0.95) | 8.42 (+0.16) |
|  | Granite Is. | ON | 84.11 (+0.91) | 95.75 (+6.21) | 8.25 (+0.16) |
| Niagara River | Weseloh Rocks | ON | 84.74 (+0.67) | 89.93 (+0.81) | 7.95 (+0.32) |
| St. Lawrence River | Île Bellechasse | QC | 91.86 (+1.86) | 90.03 (+2.41) | 7.72 (+0.24) |
|  | Île Deslauriers | QC | 89.63 (+1.18) | 86.44 (+1.18) | 7.75 (+0.14) |
|  | Strachan Is. | ON | 90.88 (+0.53) | 95.41 (+1.45) | 7.87 (+0.18) |

**Table S5**. Concentrations (ng/g, wet weight) of HBCDD, ∑_11_PBDE congeners, BDE-209 and ∑DP in herring gull eggs for each year at each study colony. Concentrations are shown as means when multiple egg samples were analyzed or as single concentration when one pooled sample was analyzed. Number of egg pools analyzed for HFRs are shown with the number in brackets representing the number of egg pools analyzed for HBCDD only since this compound could not be quantified in some egg samples.

| **Region** | **Colony** | **Province/**  **State** | **Year** | **No. Egg Pools Analyzed** | **Mean HBCDD** | **Mean ∑_11_PBDE** | **Mean BDE-209** | **Mean ∑DP** |
| --- | --- | --- | --- | --- | --- | --- | --- | --- |
| Arctic | East Bay | NU | 2010 | 5 | 2.48 | 55.83 | 3.71 | 0.18 |
|  |  |  | 2011 | 5 | 0.96 | 53.33 | 5.00 | 0.44 |
|  |  |  | 2012 | 5 | 0.72 | 44.77 | 3.67 | 0.39 |
|  |  |  | 2013 | 5 | 2.74 | 64.74 | 9.82 | 0.56 |
|  |  |  | 2015 | 5 | 2.20 | 72.79 | 4.17 | 0.64 |
|  |  |  | 2017 | 4 | 3.73 | 73.97 | 7.07 | 0.77 |
|  |  |  | 2019 | 5 | 0.81 | 50.05 | 2.19 | 0.28 |
|  |  |  | 2022 | 5 | 1.18 | 56.81 | 2.66 | 0.47 |
|  |  |  | 2023 | 5 | 2.90 | 97.51 | 2.90 | 0.44 |
| Arctic | Great Slave Lake | NT | 2010 | 5 | 10.71 | 78.94 | 28.50 | 0.62 |
|  |  |  | 2011 | 5 | 3.97 | 101.84 | 9.96 | 1.06 |
|  |  |  | 2012 | 5 (2) | 3.45 | 186.26 | 9.35 | 0.27 |
|  |  |  | 2015 | 5 | 3.89 | 38.66 | 2.88 | 0.05 |
|  |  |  | 2017 | 5 | 4.03 | 82.02 | 2.37 | 0.15 |
|  |  |  | 2022 | 1 | 2.01 | 372.47 | 7.66 | 0.30 |
| Atlantic | Gull Is. | NL | 2008 | 5 | 2.12 | 91.12 | 9.20 | 0.97 |
|  |  |  | 2009 | 5 | 0.82 | 144.11 | 17.52 | 0.50 |
|  |  |  | 2010 | 5 | 9.32 | 175.87 | 21.41 | 1.27 |
|  |  |  | 2011 | 5 | 20.90 | 155.31 | 23.72 | 4.98 |
|  |  |  | 2012 | 5 (3) | 10.52 | 107.97 | 13.44 | 1.69 |
|  |  |  | 2013 | 5 | 9.07 | 136.22 | 12.06 | 2.11 |
|  |  |  | 2015 | 5 | 8.03 | 110.61 | 11.27 | 1.67 |
|  |  |  | 2017 | 5 | 6.56 | 72.74 | 10.20 | 0.37 |
|  |  |  | 2019 | 5 | 8.15 | 92.03 | 19.18 | 0.74 |
|  |  |  | 2022 | 5 | 3.91 | 131.43 | 18.66 | 0.85 |
|  |  |  | 2023 | 5 | 8.67 | 96.62 | 12.67 | 0.32 |
| Atlantic | Kent Is. | NB | 2008 | 5 | 6.66 | 221.95 | 12.57 | 0.67 |
|  |  |  | 2010 | 5 | 3.92 | 119.98 | 6.74 | 2.07 |
|  |  |  | 2011 | 5 | 11.43 | 189.22 | 11.01 | 3.23 |
|  |  |  | 2012 | 5 (4) | 3.98 | 221.64 | 17.01 | 1.31 |
|  |  |  | 2013 | 5 | 7.65 | 123.21 | 8.48 | 0.88 |
|  |  |  | 2015 | 5 | 7.54 | 191.72 | 15.93 | 1.97 |
|  |  |  | 2017 | 5 | 2.87 | 62.52 | 3.82 | 0.23 |
|  |  |  | 2019 | 5 | 1.68 | 49.64 | 4.60 | 0.19 |
|  |  |  | 2022 | 5 | 2.63 | 53.17 | 4.09 | 2.39 |
|  |  |  | 2023 | 5 | 2.55 | 65.00 | 5.90 | 0.49 |
| Lake Erie | Middle Is. | ON | 2008 | 1 | 2.70 | 153.42 | 12.49 | 1.69 |
|  |  |  | 2009 | 1 | 3.24 | 265.48 | 8.92 | 1.12 |
|  |  |  | 2010 | 1 | 11.93 | 185.50 | 21.63 | 4.24 |
|  |  |  | 2011 | 1 | 3.31 | 161.46 | 1.84 | 0.07 |
|  |  |  | 2012 | 1 | 8.14 | 219.54 | 12.40 | 6.15 |
|  |  |  | 2013 | 1 | 4.46 | 163.33 | 12.20 | 5.30 |
|  |  |  | 2014 | 1 | 11.10 | 186.29 | 9.72 | 71.60 |
|  |  |  | 2015 | 1 | 7.68 | 205.18 | 23.10 | 21.49 |
|  |  |  | 2016 | 1 | 15.00 | 324.05 | 27.00 | 3.64 |
|  |  |  | 2017 | 3 | 4.00 | 148.37 | 4.72 | 2.73 |
|  |  |  | 2018 | 1 | 5.09 | 322.42 | 6.70 | 1.71 |
|  |  |  | 2019 | 1 | 3.53 | 188.13 | 6.66 | 6.81 |
|  |  |  | 2021 | 1 | 1.17 | 159.34 | 7.34 | 3.38 |
|  |  |  | 2022 | 5 | 1.99 | 199.82 | 7.71 | 1.91 |
|  |  |  | 2023 | 1 | 3.23 | 190.41 | 4.68 | 0.08 |
| Lake Erie | Port Colborne | ON | 2008 | 1 | 1.41 | 114.83 | 6.34 | 0.72 |
|  |  |  | 2009 | 1 | 0.02 | 277.76 | 7.84 | 1.53 |
|  |  |  | 2010 | 1 | 27.53 | 226.07 | 11.76 | 2.36 |
|  |  |  | 2011 | 1 | 6.68 | 173.08 | 1.79 | 0.04 |
|  |  |  | 2012 | 1 | 9.66 | 316.72 | 23.40 | 4.94 |
|  |  |  | 2013 | 1 | 2.66 | 154.93 | 7.63 | 2.80 |
|  |  |  | 2014 | 1 | 4.24 | 248.83 | 14.50 | 2.54 |
|  |  |  | 2015 | 1 | 8.03 | 310.44 | 32.40 | 7.90 |
|  |  |  | 2016 | 1 | 7.15 | 302.83 | 33.60 | 4.32 |
|  |  |  | 2017 | 3 | 6.07 | 235.12 | 7.67 | 1.11 |
|  |  |  | 2018 | 1 | 3.84 | 230.13 | 8.52 | 1.21 |
|  |  |  | 2019 | 1 | 4.53 | 359.52 | 11.50 | 2.06 |
|  |  |  | 2022 | 5 | 2.08 | 157.61 | 4.89 | 1.42 |
|  |  |  | 2023 | 1 | 6.38 | 134.12 | 5.31 | 1.77 |
| Lake Huron | Chantry Is. | ON | 2008 | 1 | 6.78 | 132.90 | 10.86 | 2.28 |
|  |  |  | 2009 | 1 | 8.36 | 355.98 | 28.53 | 3.61 |
|  |  |  | 2010 | 1 | 31.51 | 434.80 | 14.56 | 2.92 |
|  |  |  | 2011 | 1 | 14.67 | 192.56 | 9.84 | 5.07 |
|  |  |  | 2012 | 1 | 16.50 | 460.44 | 21.50 | 3.52 |
|  |  |  | 2013 | 1 | 6.49 | 210.23 | 9.28 | 2.19 |
|  |  |  | 2014 | 1 | 14.10 | 238.61 | 21.10 | 4.32 |
|  |  |  | 2015 | 1 | 9.82 | 343.53 | 23.00 | 4.84 |
|  |  |  | 2016 | 1 | 17.00 | 246.96 | 26.40 | 4.34 |
|  |  |  | 2017 | 3 | 7.22 | 195.96 | 14.61 | 2.02 |
|  |  |  | 2018 | 1 | 9.81 | 257.36 | 16.80 | 1.44 |
|  |  |  | 2019 | 1 | 5.69 | 179.03 | 11.70 | 1.21 |
|  |  |  | 2022 | 5 | 6.75 | 164.85 | 9.19 | 2.40 |
|  |  |  | 2023 | 1 | 5.53 | 219.54 | 12.30 | 1.84 |
| Lake Huron | Double Is. | ON | 2008 | 1 | 7.30 | 213.03 | 9.02 | 1.28 |
|  |  |  | 2009 | 1 | 11.41 | 240.28 | 22.20 | 1.69 |
|  |  |  | 2010 | 1 | 28.91 | 248.38 | 19.52 | 1.74 |
|  |  |  | 2011 | 1 | 17.90 | 304.96 | 4.31 | 4.12 |
|  |  |  | 2012 | 1 | 13.50 | 231.40 | 18.40 | 3.42 |
|  |  |  | 2013 | 1 | 0.31 | 219.75 | 12.10 | 2.77 |
|  |  |  | 2014 | 1 | 17.60 | 217.40 | 17.10 | 25.81 |
|  |  |  | 2015 | 1 | 11.60 | 353.24 | 24.10 | 10.49 |
|  |  |  | 2016 | 1 | 12.20 | 274.61 | 30.70 | 3.55 |
|  |  |  | 2017 | 3 | 10.41 | 229.64 | 22.07 | 2.08 |
|  |  |  | 2018 | 1 | 11.70 | 627.03 | 16.00 | 4.53 |
|  |  |  | 2019 | 1 | 7.17 | 376.96 | 23.20 | 1.87 |
|  |  |  | 2022 | 5 | 10.75 | 300.19 | 10.84 | 1.72 |
|  |  |  | 2023 | 1 | 8.57 | 309.98 | 19.70 | 1.77 |
| Lake Ontario | Hamilton H. | ON | 2008 | 1 | 2.77 | 267.03 | 7.71 | 2.06 |
|  |  |  | 2009 | 1 | 5.62 | 385.16 | 11.53 | 8.20 |
|  |  |  | 2010 | 1 | 18.94 | 449.72 | 11.65 | 8.35 |
|  |  |  | 2011 | 1 | 1.66 | 355.24 | 1.98 | 4.74 |
|  |  |  | 2012 | 1 | 10.90 | 296.39 | 7.42 | 11.53 |
|  |  |  | 2013 | 1 | 4.34 | 249.91 | 7.60 | 2.74 |
|  |  |  | 2014 | 1 | 9.00 | 463.67 | 17.10 | 5.76 |
|  |  |  | 2015 | 5 | 6.04 | 524.47 | 25.74 | 5.59 |
|  |  |  | 2016 | 1 | 8.30 | 324.46 | 17.50 | 2.82 |
|  |  |  | 2017 | 3 | 7.38 | 344.86 | 7.30 | 1.27 |
|  |  |  | 2018 | 1 | 5.87 | 426.55 | 8.31 | 1.32 |
|  |  |  | 2019 | 5 | 4.69 | 334.27 | 10.34 | 1.20 |
|  |  |  | 2021 | 1 | 5.58 | 330.14 | 7.72 | 1.67 |
|  |  |  | 2023 | 1 | 10.70 | 249.73 | 9.48 | 3.08 |
| Lake Ontario | Snake Is. | ON | 2008 | 1 | 2.01 | 200.98 | 4.38 | 0.50 |
|  |  |  | 2009 | 1 | 0.02 | 312.43 | 4.30 | 0.87 |
|  |  |  | 2010 | 1 | 56.55 | 289.02 | 0.29 | 0.03 |
|  |  |  | 2011 | 1 | 19.94 | 294.61 | 1.17 | 0.05 |
|  |  |  | 2012 | 1 | 14.40 | 364.45 | 12.90 | 2.64 |
|  |  |  | 2013 | 1 | 4.02 | 205.85 | 1.44 | 1.55 |
|  |  |  | 2014 | 1 | 5.82 | 186.51 | 13.20 | 2.95 |
|  |  |  | 2015 | 1 | 8.00 | 315.62 | 33.10 | 7.93 |
|  |  |  | 2016 | 1 | 5.24 | 188.46 | 9.92 | 0.73 |
|  |  |  | 2017 | 2 | 5.52 | 188.64 | 4.66 | 0.40 |
|  |  |  | 2018 | 1 | 5.80 | 262.31 | 12.50 | 0.60 |
|  |  |  | 2019 | 1 | 3.61 | 178.39 | 10.00 | 1.00 |
|  |  |  | 2021 | 1 | 2.36 | 130.87 | 5.52 | 0.78 |
|  |  |  | 2022 | 5 | 3.55 | 281.44 | 7.37 | 1.00 |
|  |  |  | 2023 | 1 | 2.35 | 146.90 | 7.52 | 0.07 |
| Lake Ontario | Toronto H. | ON | 2008 | 1 | 6.96 | 339.10 | 29.15 | 3.24 |
|  |  |  | 2009 | 1 | 8.14 | 526.94 | 34.81 | 5.48 |
|  |  |  | 2010 | 5 (4) | 7.42 | 392.68 | 18.32 | 4.01 |
|  |  |  | 2011 | 5 | 10.76 | 388.57 | 26.53 | 7.85 |
|  |  |  | 2012 | 5 | 0.23 | 277.23 | 20.82 | 9.09 |
|  |  |  | 2013 | 5 | 8.43 | 274.49 | 10.50 | 3.85 |
|  |  |  | 2014 | 1 | 14.30 | 146.33 | 51.90 | 8.23 |
|  |  |  | 2015 | 1 | 9.10 | 451.36 | 34.90 | 9.29 |
|  |  |  | 2016 | 1 | 9.97 | 293.75 | 28.50 | 4.06 |
|  |  |  | 2017 | 1 | 19.90 | 425.92 | 14.40 | 2.80 |
|  |  |  | 2018 | 1 | 11.00 | 453.82 | 22.20 | 4.85 |
|  |  |  | 2019 | 1 | 6.42 | 359.10 | 14.03 | 3.98 |
|  |  |  | 2021 | 1 | 7.84 | 280.20 | 8.13 | 2.36 |
| Lake Superior | Agawa Rocks | ON | 2008 | 1 | 7.53 | 167.49 | 9.30 | 4.40 |
|  |  |  | 2009 | 1 | 10.18 | 467.12 | 27.43 | 3.43 |
|  |  |  | 2010 | 5 | 10.04 | 234.97 | 27.82 | 6.45 |
|  |  |  | 2011 | 5 (4) | 5.41 | 161.72 | 8.44 | 5.48 |
|  |  |  | 2012 | 5 | 10.98 | 275.62 | 16.27 | 3.48 |
|  |  |  | 2013 | 5 | 7.83 | 323.70 | 13.68 | 3.24 |
|  |  |  | 2014 | 1 | 12.00 | 262.65 | 23.40 | 13.13 |
|  |  |  | 2015 | 5 | 13.26 | 290.18 | 38.32 | 14.97 |
|  |  |  | 2016 | 1 | 10.90 | 343.36 | 19.50 | 5.71 |
|  |  |  | 2017 | 3 | 9.01 | 185.26 | 11.41 | 5.75 |
|  |  |  | 2018 | 1 | 11.90 | 499.33 | 26.40 | 2.11 |
|  |  |  | 2019 | 5 | 11.30 | 209.93 | 16.30 | 2.26 |
|  |  |  | 2021 | 1 | 5.49 | 210.97 | 8.31 | 1.24 |
|  |  |  | 2022 | 5 | 9.99 | 406.16 | 13.88 | 2.27 |
|  |  |  | 2023 | 1 | 4.70 | 191.17 | 10.30 | 4.20 |
| Lake Superior | Granite Is. | ON | 2008 | 1 | 7.31 | 307.61 | 22.17 | 3.22 |
|  |  |  | 2009 | 1 | 5.63 | 342.57 | 26.53 | 2.79 |
|  |  |  | 2010 | 1 | 45.02 | 312.58 | 21.27 | 5.52 |
|  |  |  | 2011 | 1 | 3.57 | 315.92 | 1.42 | 2.78 |
|  |  |  | 2012 | 1 | 14.10 | 429.98 | 19.00 | 4.75 |
|  |  |  | 2013 | 1 | 0.29 | 402.67 | 36.20 | 9.02 |
|  |  |  | 2014 | 1 | 27.10 | 440.72 | 44.60 | 7.02 |
|  |  |  | 2015 | 1 | 17.20 | 496.81 | 32.90 | 11.41 |
|  |  |  | 2016 | 1 | 13.30 | 507.92 | 17.80 | 1.85 |
|  |  |  | 2017 | 1 | 18.20 | 346.01 | 13.80 | 2.07 |
|  |  |  | 2018 | 1 | 21.90 | 530.59 | 16.10 | 2.38 |
|  |  |  | 2019 | 1 | 12.80 | 356.57 | 26.90 | 1.95 |
|  |  |  | 2020 | 1 | 16.80 | 416.68 | 25.53 | 2.29 |
|  |  |  | 2021 | 1 | 10.20 | 335.11 | 16.10 | 1.24 |
|  |  |  | 2022 | 5 | 8.81 | 333.35 | 17.52 | 2.61 |
|  |  |  | 2023 | 1 | 22.90 | 317.28 | 16.50 | 1.13 |
| Niagara R. | Weseloh Rocks | ON | 2008 | 1 | 2.60 | 203.30 | 36.78 | 3.37 |
|  |  |  | 2009 | 1 | 3.82 | 231.12 | 22.06 | 5.94 |
|  |  |  | 2010 | 1 | 20.93 | 221.23 | 16.18 | 8.03 |
|  |  |  | 2011 | 1 | 7.67 | 218.11 | 1.69 | 12.12 |
|  |  |  | 2012 | 1 | 7.18 | 248.95 | 11.20 | 7.52 |
|  |  |  | 2013 | 1 | 3.15 | 179.29 | 6.30 | 5.76 |
|  |  |  | 2014 | 1 | 5.28 | 187.14 | 19.60 | 3.63 |
|  |  |  | 2015 | 1 | 5.36 | 374.70 | 44.50 | 15.95 |
|  | Buffalo H. | NY | 2018 | 1 | 3.96 | 298.08 | 19.00 | 4.71 |
|  |  |  | 2019 | 1 | 3.44 | 251.78 | 27.70 | 4.32 |
| St. Lawrence R. | Île Bellechasse | QC | 2008 | 5 | 6.45 | 183.63 | 31.48 | 0.61 |
|  |  |  | 2009 | 3 | 3.08 | 442.87 | 68.69 | 3.28 |
|  |  |  | 2010 | 5 | 19.89 | 281.19 | 39.44 | 4.50 |
|  |  |  | 2011 | 5 | 31.40 | 565.22 | 43.66 | 4.93 |
|  |  |  | 2012 | 5 (0) | - | 363.95 | 26.68 | 1.69 |
|  |  |  | 2013 | 5 | 18.44 | 437.72 | 49.32 | 11.74 |
|  |  |  | 2015 | 5 | 19.09 | 201.89 | 23.04 | 9.51 |
|  |  |  | 2017 | 5 | 7.16 | 202.06 | 19.12 | 2.06 |
|  |  |  | 2019 | 5 | 5.72 | 222.16 | 17.55 | 1.53 |
|  |  |  | 2022 | 5 | 4.09 | 85.25 | 8.21 | 1.22 |
|  |  |  | 2023 | 5 | 6.39 | 84.95 | 13.12 | 0.55 |
| St. Lawrence R. | Île Deslauriers | QC | 2008 | 5 | 12.89 | 629.57 | 165.08 | 1.86 |
|  |  |  | 2010 | 5 | 14.32 | 365.83 | 16.28 | 2.76 |
|  |  |  | 2011 | 5 | 28.38 | 755.69 | 23.94 | 4.16 |
|  |  |  | 2012 | 5 (0) | - | 697.83 | 28.82 | 1.92 |
|  |  |  | 2013 | 4 | 11.64 | 473.54 | 29.48 | 4.69 |
|  |  |  | 2015 | 5 | 11.20 | 587.32 | 37.90 | 4.37 |
|  |  |  | 2017 | 3 | 81.08 | 846.95 | 79.90 | 8.77 |
|  |  |  | 2019 | 1 | 7.39 | 585.79 | 45.50 | 2.91 |
|  |  |  | 2022 | 1 | 6.40 | 592.54 | 40.60 | 0.97 |
|  |  |  | 2023 | 1 | 14.10 | 370.04 | 50.00 | 2.62 |
| St. Lawrence R. | Strachan Is. | ON | 2008 | 1 | 7.04 | 217.35 | 11.80 | 1.09 |
|  |  |  | 2009 | 1 | 5.24 | 262.07 | 16.63 | 1.65 |
|  |  |  | 2010 | 1 | 24.55 | 240.80 | 13.08 | 2.20 |
|  |  |  | 2011 | 1 | 23.40 | 329.40 | 7.10 | 4.02 |
|  |  |  | 2012 | 1 | 13.90 | 206.60 | 23.60 | 3.01 |
|  |  |  | 2013 | 1 | 6.38 | 180.13 | 16.10 | 2.91 |
|  |  |  | 2014 | 1 | 9.10 | 192.93 | 18.00 | 1.49 |
|  |  |  | 2015 | 1 | 7.05 | 187.40 | 18.60 | 2.89 |
|  |  |  | 2016 | 1 | 8.57 | 210.13 | 16.10 | 1.66 |
|  |  |  | 2017 | 3 | 8.67 | 170.42 | 10.55 | 0.80 |
|  |  |  | 2018 | 1 | 4.84 | 174.32 | 9.78 | 0.63 |
|  |  |  | 2019 | 1 | 5.06 | 156.42 | 25.60 | 1.23 |
|  |  |  | 2022 | 5 | 5.68 | 131.51 | 146.56 | 4.71 |
|  |  |  | 2023 | 1 | 4.24 | 109.40 | 100.00 | 3.18 |

Table S6. AIC and likelihood‐ratio tests comparing full models vs null models (with or without Region as a factor) to determine if Region was a significant predictor of halogenated flame retardant (HFR) concentrations in eggs.
Null Model: $y_{ijk}=\beta_{0}+u_{ij}+v_{k}+\epsilon_{ijk}$
Full Model: $y_{ijk}=\beta_{0}+\beta_{Region(i)}+u_{ij}+v_{k}+\epsilon_{ijk}$

where, *y_ijk_*= ln[HFR] for colony *j* in Region *i* during Year *k*; β_0_ = overall intercept; β*i* = fixed effect for Region *i*, *u_ij_* = random intercept for colony *j* (nested within Region *i*), *v_k_* = random intercept for Year *k*, and *ε_ijk_* = residual error.

**R syntax**:

Reduced Model: ln([HFR]) ~ 1 + (1 | Region:Colony) + (1 | Year)

Full Model: ln([HFRs]) ~ Region + (1 | Region:Colony) + (1 | Year)

|  | # parameters | AIC | Log Likelihood | Deviance | χ^2^ | df | Pr(>χ^2^) |
| --- | --- | --- | --- | --- | --- | --- | --- |
| **Σ_11_PBDE** |  |  |  |  |  |  |  |
| Null model | 4 | 968.0 | -480.0 | 960.0 |  |  |  |
| Full model | 7 | 945.6 | -465.8 | 931.6 | 28.4 | 3 | < 0.0001 |
|  |  |  |  |  |  |  |  |
| **BDE 209** |  | |  |  |  |  |  |
| Null model | 4 | 1353.5 | -672.8 | 1345.5 |  |  |  |
| Full model | 7 | 1336.2 | -661.1 | 1322.2 | 23.4 | 3 | < 0.0001 |
|  |  |  |  |  |  |  | |
| **HBCDD** |  |  |  |  |  |  | |
| Null model | 4 | 1576.4 | -784.2 | 1568.4 |  |  |  |
| Full model | 7 | 1566.6 | -776.3 | 1552.6 | 15.8 | 3 | 0.00125 |
|  |  |  |  |  |  |  |  |
| **Sum DP** |  | |  |  |  |  |  |
| Null model | 4 | 1598.2 | -795.1 | 1590.2 |  |  |  |
| Full model | 7 | 1572.5 | -779.3 | 1558.5 | 31.6 | 3 | < 0.0001 |

Table S7a. Random effects of Linear Mixed Models comparing natural logged Σ_11_PBDE in herring gull eggs among Regions (Atlantic, Great Lakes, St. Lawrence River, Arctic). Model: $y_{ijk}=\beta_{0}+\beta_{Region(i)}+u_{ij}+v_{k}+\epsilon_{ijk}$

Σ_11_PBDE was the sum of BDEs 17, 28, 47, 49, 66, 85, 99, 100, 138, 153, and 183)

| Random effects: |  | |  |  |
| --- | --- | --- | --- | --- |
| Groups | Name | Variance | Std.Dev. | % Variance |
| Region:Colony | (Intercept) | 0.081 | 0.284 | 18.4 |
| Year | (Intercept) | 0.031 | 0.177 | 7.0 |
| Residual | 0.329 | | 0.574 | 74.6 |

| Table S7b. Estimated marginal means, using Satterthwaite’s approximation of degrees of freedom, for fixed effects of Linear Mixed Models comparing natural logged Σ_11_PBDE in herring gull eggs among Regions (Atlantic, Great Lakes, St. Lawrence River, Arctic). Model: $y_{ijk}=\beta_{0}+\beta_{Region(i)}+u_{ij}+v_{k}+\epsilon_{ijk}$  Fixed effects: |  |  |  |
| --- | --- | --- | --- |

| Region | emmean | SE | df | lower.CL | upper.CL | t.ratio | p.value |
| --- | --- | --- | --- | --- | --- | --- | --- |
| Atlantic | 4.55 | 0.21 | 12.23 | 4.08 | 5.02 | 21.21 | <0.001 |
| Great Lakes | 5.58 | 0.11 | 18.63 | 5.35 | 5.80 | 51.14 | <0.001 |
| St Lawrence River | 5.64 | 0.18 | 13.88 | 5.26 | 6.03 | 31.13 | <0.001 |
| Arctic | 4.05 | 0.22 | 13.38 | 3.57 | 4.52 | 18.42 | <0.001 |
| Overall mean |  |  |  |  |  |  |  |

Table S8a. Random effects of Linear Mixed Models comparing natural logged BDE 209 in herring gull eggs among Regions (Atlantic, Great Lakes, St. Lawrence River, Arctic). Model: $y_{ijk}=\beta_{0}+\beta_{Region(i)}+u_{ij}+v_{k}+\epsilon_{ijk}$

| Random effects: |  | |  |  |  |
| --- | --- | --- | --- | --- | --- |
| Groups | Name | Variance | Std.Dev. | % Variance | |
| Region:Colony | (Intercept) | 0.110 | 0.331 | 12.6 | |
| Year | (Intercept) | 0.037 | 0.193 | 4.2 | |
| Residual | 0.724 | | 0.851 | 83.1 | |

Table S8b. Estimated marginal means, using Satterthwaite’s approximation of degrees of freedom, for fixed effects of Linear Mixed Models comparing natural logged BDE 209 in herring gull eggs among Regions (Atlantic, Great Lakes, St. Lawrence River, Arctic). Model: $y_{ijk}=\beta_{0}+\beta_{Region(i)}+u_{ij}+v_{k}+\epsilon_{ijk}$

| Fixed effects: |  |  |  |
| --- | --- | --- | --- |

| Region | emmean | SE | df | lower.CL | upper.CL | t.ratio | p.value |
| --- | --- | --- | --- | --- | --- | --- | --- |
| Atlantic | 2.19 | 0.255 | 11.2 | 1.63 | 2.75 | 8.60 | <.0001 |
| Great Lakes | 2.5 | 0.131 | 17.7 | 2.22 | 2.77 | 19.04 | <.0001 |
| St Lawrence River | 3.37 | 0.218 | 13.1 | 2.9 | 3.84 | 15.47 | <.0001 |
| Arctic | 1.29 | 0.264 | 12.8 | 0.72 | 1.86 | 4.89 | 0.0003 |

Table S9a. Random effects of Linear Mixed Models comparing natural logged HBCDD in herring gull eggs among Regions (Atlantic, Great Lakes, St. Lawrence River, Arctic). Model: $y_{ijk}=\beta_{0}+\beta_{Region(i)}+u_{ij}+v_{k}+\epsilon_{ijk}$

| Random effects: |  | |  |  |
| --- | --- | --- | --- | --- |
| Groups | Name | Variance | Std.Dev. | % Variance |
| Region:Colony | (Intercept) | 0.162 | 0.403 | 8.8 |
| Year | (Intercept) | 0.464 | 0.681 | 25.2 |
| Residual | 1.217 | | 1.103 | 66.0 |

Table S9b. Estimated marginal means, using Satterthwaite’s approximation of degrees of freedom, for fixed effects of Linear Mixed Models comparing natural logged HBCDD in herring gull eggs among Regions (Atlantic, Great Lakes, St. Lawrence River, Arctic). Model: $y_{ijk}=\beta_{0}+\beta_{Region(i)}+u_{ij}+v_{k}+\epsilon_{ijk}$

| Fixed effects: |  |  |  |
| --- | --- | --- | --- |

| Region | emmean | SE | df | lower.CL | upper.CL | t.ratio | p.value |
| --- | --- | --- | --- | --- | --- | --- | --- |
| Atlantic | 1.25 | 0.355 | 16.4 | 0.50 | 2.00 | 3.51 | 0.003 |
| Great Lakes | 1.77 | 0.230 | 24.2 | 1.30 | 2.25 | 7.69 | <.0001 |
| St Lawrence River | 2.12 | 0.317 | 21.1 | 1.46 | 2.78 | 6.69 | <.0001 |
| Arctic | 0.35 | 0.368 | 18.8 | -0.42 | 1.12 | 0.96 | 0.351 |

Table S10a. Random effects of Linear Mixed Models comparing natural logged sum DP in herring gull eggs among Regions (Atlantic, Great Lakes, St. Lawrence River, Arctic). Model$: y_{ijk}=\beta_{0}+\beta_{Region(i)}+u_{ij}+v_{k}+\epsilon_{ijk}$

| Groups | Name | Variance | Std.Dev. | % Variance |
| --- | --- | --- | --- | --- |
| Region:Colony | (Intercept) | 0.161 | 0.401 | 11.0 |
| Year | (Intercept) | 0.171 | 0.414 | 11.7 |
| Residual | 1.135 | | 1.066 | 77.4 |

Table S10b. Estimated marginal means, using Satterthwaite’s approximation of degrees of freedom, for fixed effects of Linear Mixed Models comparing natural logged sum DP in herring gull eggs among Regions (Atlantic, Great Lakes, St. Lawrence River, Arctic). Model: $y_{ijk}=\beta_{0}+\beta_{Region(i)}+u_{ij}+v_{k}+\epsilon_{ijk}$

| Fixed effects: |  |  |  |
| --- | --- | --- | --- |

| Region | emmean | SE | df | lower.CL | upper.CL | t.ratio | p.value |
| --- | --- | --- | --- | --- | --- | --- | --- |
| Atlantic | -0.37 | 0.323 | 12.8 | -1.07 | 0.33 | -1.14 | 0.2765 |
| Great Lakes | 0.92 | 0.183 | 22.9 | 0.54 | 1.30 | 5.03 | <.0001 |
| St Lawrence River | 0.84 | 0.280 | 15.7 | 0.24 | 1.43 | 2.99 | 0.0087 |
| Arctic | -1.96 | 0.335 | 14.7 | -2.68 | -1.25 | -5.86 | <.0001 |

Figure S1. Boxplots of median, 95% confidence intervals around the median, 25^th^ and 75^th^ percentiles (interquartile range) and extreme values within +/- 1.5 × interquartile range, for a) Σ_11_PBDEs, b) BDE209, c) HBCDD, and d) sum DP (ng/g ww) in herring gull eggs among Regions (Great Lakes, Arctic, Atlantic and St Lawrence River). Horizontal dotted lines is the overall mean.


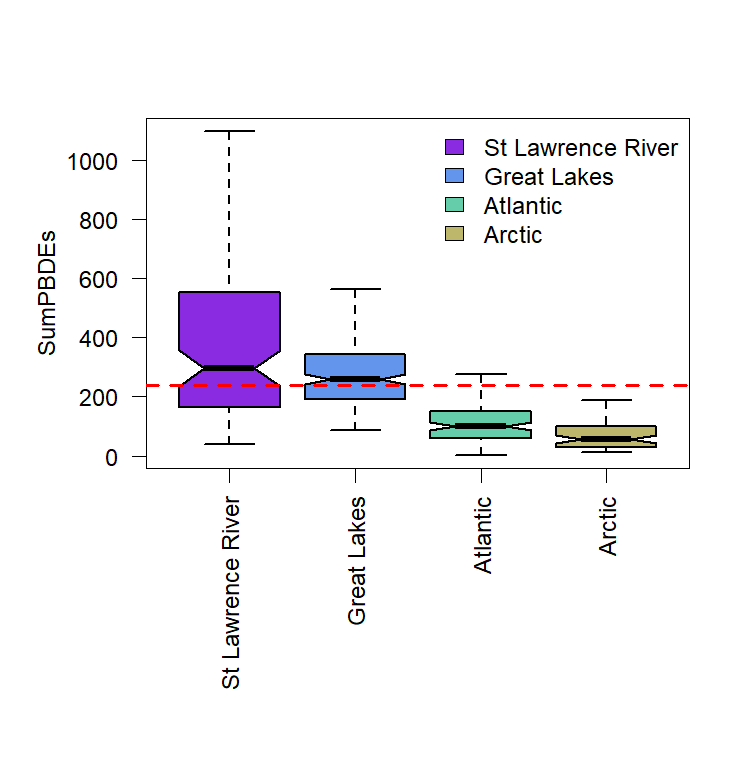


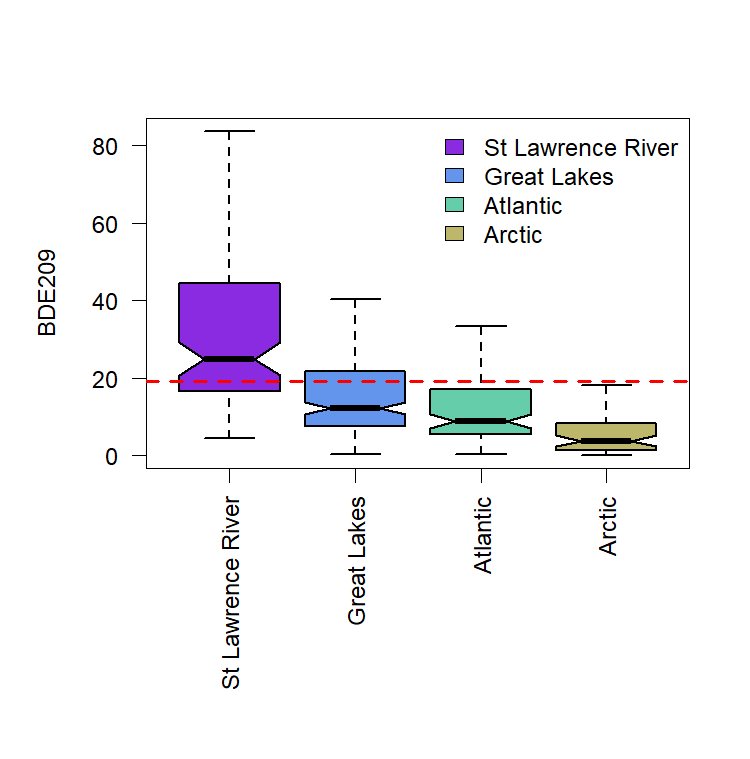


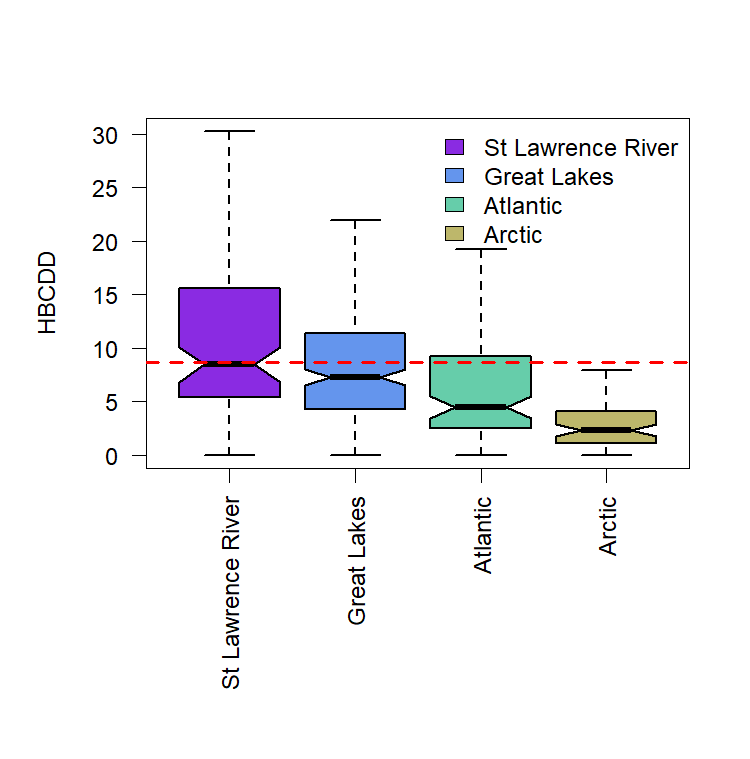


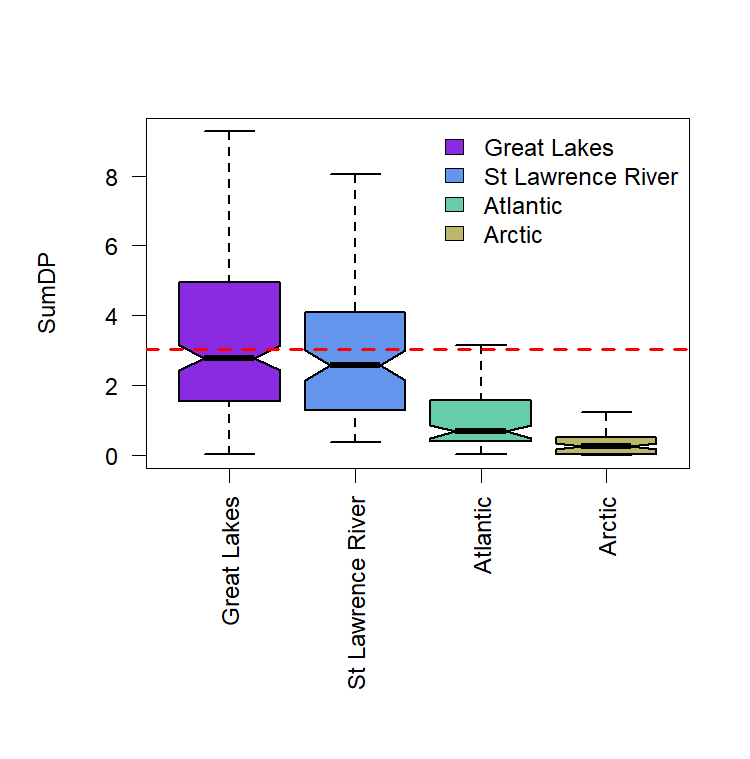


**R code (4.4.2)**

***Statistics***

# Spatial comparisons of HFRs among colonies

# Install required packages
packages <- c("lmerTest", "emmeans", "dplyr", "readr", "performance", "car")
install_if_missing <- function(pkg) {
  if (!requireNamespace(pkg, quietly = TRUE)) install.packages(pkg)
}
invisible(lapply(packages, install_if_missing))
# Load packages
library(lmerTest)
library(emmeans)
library(dplyr)
library(readr)
library(performance)
library(car)
# Load updated data
data <- read_csv("HFR_by_locations_all_data_with_log.csv")

# Note Location in the datafile is Colony in the text

# Set sum-to-zero contrasts instead of first level of treatment; i.e. compare treatments to overall mean
options(contrasts = c("contr.sum", "contr.poly"))

# Fit the linear mixed model
model <- lmer(log_SumPBDEs ~ Region + (1 | Region:Location) + (1 | Year), data = data)
full_model_ML <- lmer(log_SumPBDEs ~ Region + (1 | Region:Location) + (1 | Year), data = data, REML = FALSE)
null_model_ML <- lmer(log_SumPBDEs ~ 1 + (1 | Region:Location) + (1 | Year), data = data, REML = FALSE)
anova_result <- anova(null_model_ML, full_model_ML, test = "LRT")
print(anova_result)

# Model summary
summary_output <- summary(model)

# Export fixed effects
fixed_effects <- summary_output$coefficients
write_csv(as.data.frame(fixed_effects), "fixed_effects_summary_SumPBDEs.csv")

# Export random effects
random_effects <- as.data.frame(VarCorr(model))
write_csv(random_effects, "random_effects_summary_SumPBDEs.csv")

# Export ICC
icc_value <- performance::icc(model)
write_csv(as.data.frame(icc_value), "icc_summary_SumPBDEs.csv")

# ANOVA with Satterthwaite's method
anova_output <- anova(model)
write_csv(as.data.frame(anova_output), "anova_summary_SumPBDEs.csv")

# Estimated marginal means (back-transformed to original metric) for frequentist purists like myself
emm <- emmeans(model, ~ Region, type = "response")
emm_summary <- summary(emm, infer = TRUE)
write_csv(as.data.frame(emm_summary), "emmeans_summary_SumPBDEs.csv")

# Optional: Print summaries to console
print(summary_output)
print(anova_output)
print(emm_summary)

***Graphs***

# 1. read & clean
df <- read.csv("HFR by locations all data.csv", stringsAsFactors = FALSE)
names(df) <- trimws(names(df)) # strip stray spaces in header
df$Region <- trimws(df$Region) # strip stray spaces in Region

# 2. choose metric
metric <- "HBCDD" # must match exactly a column name in your print(names(df))

# 3. compute medians by Region via split + sapply
loc_meds <- sapply(
 split(df[[metric]], df$Region),
 function(x) median(x, na.rm = TRUE)
)

# 4. inspect
print(sort(loc_meds, decreasing = TRUE))

# 5. build the ordered factor (highest median → left)
ordered_levels <- names(loc_meds)[ order(loc_meds, decreasing = TRUE) ]
df$Region <- factor(df$Region, levels = ordered_levels)

# 6. build Region colour lookup

# create colour palette with as many colours as unique Regions

palette4 <- setNames(

c("blueviolet","cornflowerblue","mediumaquamarine","darkkhaki")[seq_along(ordered_levels)],

ordered_levels

)

# 7. expand bottom margin
par(mar = c(10, 5, 4, 2) + 0.1)

# 8. draw notched, no-outlier boxplots

boxplot(

stats::as.formula(paste(metric, "~ Region")),

data = df,

notch = TRUE,

outline = FALSE,

lwd = 1.5,

col = palette4[ df$Region |> levels() ],

las = 2,

xlab = "",

ylab = metric,

main = ""

)

mean_score <- mean(df$HBCDD, na.rm = TRUE)

abline(h = mean_score,
 lty = "dashed", # or lty = 2
 lwd = 2,

col = "red")
# 9. legend
legend(
 "topright",
 legend = names(palette4),
 fill = palette4,
 bty = "n"
)
